# Radiotherapy combined with immunotherapy: the dawn of cancer treatment

**DOI:** 10.1038/s41392-022-01102-y

**Published:** 2022-07-29

**Authors:** Zengfu Zhang, Xu Liu, Dawei Chen, Jinming Yu

**Affiliations:** 1 Department of Radiation Oncology, Shandong University Cancer Center, Yantai Road, No. 2999, Jinan, Shandong China; 2 Department of Radiation Oncology, Shandong Cancer Hospital and Institute, Shandong First Medical University and Shandong Academy of Medical Sciences, Jiyan Road, No. 440, Jinan, Shandong China

**Keywords:** Cancer microenvironment, Cancer therapy

## Abstract

Radiotherapy (RT) is delivered for purposes of local control, but can also exert systemic effect on remote and non-irradiated tumor deposits, which is called abscopal effect. The view of RT as a simple local treatment has dramatically changed in recent years, and it is now widely accepted that RT can provoke a systemic immune response which gives a strong rationale for the combination of RT and immunotherapy (iRT). Nevertheless, several points remain to be addressed such as the interaction of RT and immune system, the identification of the best schedules for combination with immunotherapy (IO), the expansion of abscopal effect and the mechanism to amplify iRT. To answer these crucial questions, we roundly summarize underlying rationale showing the whole immune landscape in RT and clinical trials to attempt to identify the best schedules of iRT. In consideration of the rarity of abscopal effect, we propose that the occurrence of abscopal effect induced by radiation can be promoted to 100% in view of molecular and genetic level. Furthermore, the “radscopal effect” which refers to using low-dose radiation to reprogram the tumor microenvironment may amplify the occurrence of abscopal effect and overcome the resistance of iRT. Taken together, RT could be regarded as a trigger of systemic antitumor immune response, and with the help of IO can be used as a radical and systemic treatment and be added into current standard regimen of patients with metastatic cancer.

## Introduction

Radiotherapy (RT), a fundamental component of cancer treatment, is received by about half of all patients with cancer.^1–3^ RT is a powerful weapon for both curative purposes as well as for palliation and maintenance of the quality of life.^4,5^ As we focus on investigations of new technologies such as FLASH RT, proton RT, and carbon ion RT which aim to improve the therapeutic ratio,^6–11^ increasing evidence on immunomodulatory effects of RT casts new light on a systemic antitumor response. Recent studies have found RT may be similar to an “accelerant” by means of inducing in situ vaccination by killing tumor cells and triggering a systemic immune response.^12–20^ The most representative example is the abscopal effect: radiation on one site may cause regression of tumor at remote and distant non-irradiated sites.^21–23^ The potential systemic antitumor capacity provides a sound basis for iRT.

**Fig. 1:**
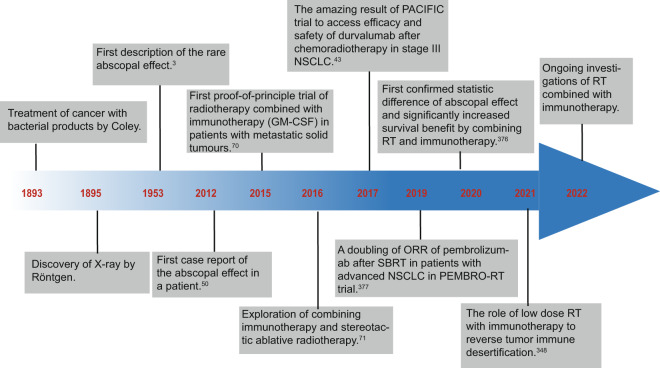
Historical timeline of some important developments regarding the iRT

In recent years, cancer immunotherapy has been considered as one of the most successful approaches in oncologic therapy, particularly with regard to immune checkpoint inhibitors (ICIs) in treating solid tumors. For some certain types of tumors such as non-small cell lung cancer (NSCLC), ICIs can significantly improve conditions of survival without interruption and induce long-term durable remission which means even patients with advanced tumor can get persistent and stable benefit from immunotherapy.^24–31^ However, the number of patients who may durably respond to IO alone just accounts for a minority of cases.^32^ It is urgent to broaden the beneficial spectrum of immunotherapy and identify appropriate patient selection. iRT can overcome the problems we mentioned above as far as possible. Thus, it is natural to combine the two treatment modalities as one is local means and another is systemic. And there are several reviews focusing on this field and presenting perspective of iRT to guide clinical trials and make the further processes (Fig. 1).^21,33–41^ Indeed, iRT has shown an exciting impact on patients with certain cancers in PACIFIC and other clinical trials.^42–48^ These convincing clinical data provide the foundation for further exploration of combination schedules.

Nevertheless, there are still many unresolved issues about iRT. First, although the abscopal effect has been quite well-known since its initial description,^3,49,50^ the underlying mechanisms of how RT influences immune cells and induces abscopal regression of tumor remain unknown. Meanwhile, according to the rarity of the abscopal effect,^51^ we should take into account how to amplify the occurrence of this phenomenon so as to expand the beneficial population. Second, the optimal patterns of iRT largely remain unresolved. The agents, sequence, dose, fractionation, and irradiated sites required further exploration.

This review will attempt to address these questions based on the latest advances in iRT in the view of rationales and clinical practice. Moreover, we will introduce “the radscopal effect” as a new concept derived from abscopal effect to show the powerful strength of low-dose radiation combined with immunotherapy.

## Development history of RT and IO

Since Röntgen discovered X-ray in 1895 and Marie Curie discovered radium and polonium, advances of radiation utilization in medicine have never slowed down. Tracing back the development history in RT, it can be summarized as two paths (physical development and biological effect) and four major eras (discovery era, orthovoltage era, megavoltage era, and ion beams era).^52,53^ During the era of discovery, which referred to the period from the discovery of X-ray to 1930, the roots of RT were established. In addition to the salient discovery by Röntgen in 1895, Becquerel reported the phenomenon of radioactivity in 1896 and in 1898 the Curies isolated radium, which lay the foundation of RT. Indeed, a patient with breast cancer received the treatment of RT using X-ray in 1896.^54^ Although X-rays were used in the clinic, biological effects and mechanisms still remain unclear. Regaud and Coutard found the advantages of fractionated therapy in the research of delivering the total radiation dose and their studies promoted the understanding of X-ray’s biological characteristics.^55^ Another crucial discovery is a practical X-ray tube which can promote the delivery of X-rays with higher-energy (180–200 kV) by Coolidge.^56^ The orthovoltage era is from 1930 to 1950 which is a booming and transitional period of physical and machine developments. The cyclotron was developed by Widerøe in 1927 and invented in 1930 by Lawrence and Livingston.^57^ Another major advance was the synchrotron, conceived by Veksler and McMillan during 1944–1945.^58^ The first synchrotron began operation in 1952 at Brookhaven National Laboratory.^59^ The megavoltage era refers to the period from about 1950 to 1985 and it seems that this era is still in progress. Cobalt teletherapy and megavoltage linear electron accelerators were considered a revolution in cancer treatment which produced radiation with high energy to treat deep tumors.^60^ In the 1960s and 1970s, electron linacs have been widely used. With the development of computer and imaging technologies, computed tomography scans and similar imaging technologies were applied to treatment planning of RT for determining tumor volumes as well as identifying normal organ anatomy. The appearance of intensity-modulated radiation therapy (IMRT) improves the radiation beams adjusted to the three-dimensional shape of the target and reduces the damage to surrounding normal tissues.^2^ In addition, image-guided radiation therapy (IGRT), using frequent pretreatment imaging to increase certainty regarding tumor location, also occurred with the technological progress. And due to the advent of these imaging technologies, stereotactic body radiotherapy (SBRT) has been widely used recent years. In the near future, FLASH RT, proton RT, and carbon ion RT may benefit more patients with reduced toxicities and improved survival outcomes.

Cancer immunotherapy has a history of more than 100 year so far. Although difficult to prove, the initiation of immunotherapy can be traced back to 2400 years ago.^61^ Nowadays, William B. Coley is widely accepted as the father of immunotherapy. The first clinical trial using IO (mixtures of *Streptococcus pyogenes* and *Serratia marcescens*) to treat tumors was designed by Coley in 1893. However, due to the lack of known mechanisms about the “Coley’s toxins” and immune system, IO has been in a slow development stage and even raises doubts about tumor immunity and its treatment. In 1959, the first cancer vaccine study conducted by Ruth and John Graham achieved success and aroused interest in this area.^62^ With the establishment of existence and the key role of T cell in adaptive immunity by Miller in 1967,^63^ cancer IO was made in rapid-fire succession. In addition to the above-mentioned development, the discovery of dendritic cells and natural killer cells, promoted the understanding of underlying mechanisms of immune system and aided in the process of cancer IO. In the 1980’s, Taniguchi et al. cloned the IL-2 and it showed promising results in clinical trials.^64,65^ Furthermore, it was approved by the US FDA in 1991 for the patients who were suffering from metastatic kidney cancer. In 1987, Brunet et al. first proved cytotoxic T-lymphocyte antigen 4 (CTLA-4) and its immune checkpoint function was proved by Jim Allison and his colleagues in 1995.^66^ Until 2010, ipilimumab as the revolutionary checkpoint inhibitor was approved by FDA for the treatment of stage IV melanoma after the definitive clinical study.^67^ Another checkpoint inhibitor, nivolumab, was approved by FDA in 2014. Atezolizumab, another checkpoint inhibitor of the programmed death 1 ligand (PD-L1) protein, was approved by FDA in 2016.

With the deepening research on biological effect of RT, it seems that combining RT and IO become an inevitable strategy to improve the outcomes of cancer treatment. In 1975, Milas et al. first proposed that the antitumor effect of local irradiation could be improved by administration of Corynebacterium granulosum or Corynebacterium parvum in C3Hf/Bu mice with a syngeneic fibrosarcoma.^68^ Besides immunosuppressive effect induced by RT, investigators realized that RT also has a potential to activate immune system of human body, which lay a solid foundation for iRT. In 2005, Demaria and his colleagues first proposed radioimmunotherapy as a novel approach to treat cancer.^69^ Following the concept of iRT, research of combining RT with various kinds of IO were flourishing. In 2012, first case report of the abscopal effect in a patient with melanoma treated with ipilimumab and RT was reported and iRT has tremendous potential to increase the occurrence of abscopal effect.^50^ The first proof-of-principle trial of RT combined with IO (GM-CSF) achieved a successful result to produce abscopal responses in patients who are suffering from metastatic solid tumors in 2015.^70^ In 2017, the amazing result from PACIFIC trial showed the significant clinical benefit and changed the modality of NSCLC. In addition, Chang et al. proposed the combination of IO with stereotactic ablative radiotherapy (SBRT) and showed the significant advantages of this strategy.^71^ Nowadays, there are hundreds of ongoing clinical trials to explore optimal combination modality of iRT and we expect more advances and further interest in this field.

## Rationale of iRT

Except the direct damage to irradiated tumor cells, ionizing radiation (IR) also induces a series of biological effects which are deemed to be systemic, immune-mediated antitumor effects.^72–80^ In fact, the immunomodulatory effects of IR are exactly theoretical basis of combination. IR can exert a potent antitumor immune response by influencing almost all steps in the cancer-immunity cycle rather than just several discrete steps with ICIs.^81,82^ These effects comprise enhancing tumor antigen release and presentation,^83,84^ promoting priming and activation of immune cells,^85,86^ increasing density of tumor-infiltrating lymphocytes,^87,88^ facilitating recognition of tumor cells by T cells and augmenting antitumor effect,^89,90^ which have been well summarized and elucidated in several reviews. Besides, in the process of activating immune system, IR also leads to proinflammatory cytokines released through cyclic GMP–AMP synthase (cGAS)-stimulator of interferon genes (STING) and other inflammatory signals.^91–93^ Furthermore, the tumor microenvironment is remodeled by cytokines and stromal, immunological, and vascular changes induced by IR and mediates an antitumor response.^94,95^ The reprogrammed tumor microenvironment induced by IR plays a role as a “game changer” to transform “cold” tumors with less immune cells infiltration into “hot” tumors with lymphocytic infiltration and provide a pre-requisite for response to ICIs. This key role of IR has been discussed and widely accepted.^96,97^

However, RT doesn’t work the way we want it to, on the contrary, the immunomodulatory effect induced by RT is a double-edged sword that not only enhances systemic antitumor immune response but also promotes immunosuppression to some extent.^35,98^ Generally, IR is deemed to inhibit the immune function of the body for its lethality to immune cells in the peripheral blood. In fact, its damage to the immune system goes far beyond that. IR also influences tumor microenvironment by increasing some inhibitory immune cells that include Treg cells and myeloid-derived suppressor cells (MDSCs).^99,100^ Besides, many studies have shown immunosuppressive cytokines are elevated after radiotherapy.

Subsequently, we will elucidate distinguished notable effects induced by IR in cancer cells, immune cells, and stromal cells, respectively, and offer a landscape of the whole immune process after radiation based on immune cells including Treg cells, neutrophils, macrophages, myeloid-derived suppressor cells, dendritic cells (DCs), natural killer cells (NKs) and other subsets of T lymphocytes.

### Effects of IR on cancer cells

Radiation damages cancer cells via two primary mechanisms: direct breakage of DNA by high-energy photons and the generation of ROS. Besides the direct impact on cancer cells, it has been proved that a series of biological events induced by DNA damage which occur in cancer cells play an important role in the immunomodulatory actions of RT.^95,101–105^ On the one hand, IR not only promotes the immunogenic cell death of cancer cells which then affects the behavior of immune cells. On account of the stress response induced by IR, some intrinsic biological processes occur in the dying cancer cells including the release of damage-associated molecular patterns (DAMPs).^34^ The release of DAMPs acts on different immune cells and exert potent immunomodulatory effects of IR which will be discussed below. On the other hand, cellular response driven by DNA damage also changes the immunogenicity of these irradiated cancer cells.^99^ Then we will discuss the relationship between DNA damage response (DDR) and the STING signaling in cancer cells which results in the formation of inflammatory microenvironment remodeling by ionizing radiation and the generation of the abscopal effect after RT.

RT is a well-documented trigger of DNA damage and this occurs via two primary mechanisms: direct breakage of DNA by high-energy photons and the generation of ROS. Under physiological conditions, DNA strand breaks can be repaired by the three central DDR kinases: DNA-dependent protein kinase (DNA-PK), ataxia telangiectasia-mutated (ATM), and ataxia telangiectasia & Rad3-related protein (ATR) which prevent progression of cells with DNA damage into mitosis and avoid exposure of DNA in the cytoplasm. These DDR kinases further facilitate two main mechanisms including non-homologous end-joining (NHEJ) and homologous recombination (HR) repair. In addition, a myriad of DDR proteins repair the genomic lesions when the DNA damage appears.^106^ However, it is very common for the disorder of DDR processes in cancer which may lead to the persistence of genomic instability.^107^ And cell cycle checkpoint disruption occurs commonly in tumor cells leading to formation of micronuclei containing DNA in the cytoplasm.^108,109^ And it is a well-established phenomenon that IR may induce the production of micronuclei in cancer cells and it is also well-known for the production of type I interferon (IFN) caused by IR.^110^ Thus, surveillance of micronuclei by cGAS builds a bridge between DDR and STING signaling pathway. For the surveillance function of micronuclei by cGAS, cytoplasmic DNA induced by IR promotes type I IFN production through cGAS–STING pathway in cancer cells and triggers subsequent innate immune signaling. One of the most important negative regulators is DNA exonuclease Trex1. Vanpouille-Box and colleagues found the key role of DNA exonuclease Trex1 in the context of RT which degrades cytoplasmic DNA preventing cGAS activation.^111^

In addition to the damaged cytoplasmic DNA, another source of immunogenic DNA is exposure of the mitochondrial genome (mtDNA) to the cytoplasm in cancer cells. mtDNA damage and reactive oxygen species (ROS) resulting from RT also provoke a series of immune responses and the mtDNA damage may be more sensitive to the disorder of DDR processes than nuclear DNA damage.^112,113^ However, the underlying mechanism of mtDNA induced by IR remains unclear. In a conclusion, all of these damaged DNA including cytoplasmic DNA and mtDNA induced by IR can be recognized by cGAS, which then oligomerizes with DNA in the form of a 2:2 complex.^114–116^ After binding to DNA, cGAS then exerts a catalytic role to promote the synthesis of the second messenger 2′3′-cyclic GMP–AMP (cGAMP).^117,118^ Binding of 2′3′-cGAMP stimulates STING and promotes the translocation to the Golgi which acivates TANK-binding kinase 1 (TBK1).^115,119,120^ TBK1 phosphorylates STING and promote the interferon regulatory factor 3 (IRF3) to translocate to the nucleus which triggers the expression of IFN-β gene. Type I IFNs induced by cGAS–STING pathway are immunomodulatory factors to destroy cancer cells as the essential link of innate and adaptive immune responses through DCs.^121,122^ Type I IFNs secreted by cancer cells can facilitate DCs maturation, increase DCs co-stimulatory molecule expression as well as enhancing DCs lymph-node migratory capacity. Moreover, it also plays a crucial role in priming CD8^+^ T cells and aiding in the following antitumor responses.

Of note, besides IRF-3, both canonical and non-canonical NF-κB pathways play a crucial role in cGAS-STING induced type I IFN expression in this context of RT. Generally, NF-κB is activated through canonical and non-canonical pathways. The canonical pathway mediates the activation of NF-κB1 p50, RELA and c-REL whereas the non-canonical NF-κB pathway selectively activates p100-sequestered NF-κB members, predominantly NF-κB2 p52 and RELB. In addition to the generation of ROS, the signaling pathways of IR-induced DNA damages also activate the NF-κB pathway. Radiation-induced NF-κB is mostly mediated via IKK-dependent canonical pathway.^123^ IFN-β largely depends on the facilitation effect of the enhanceosome and canonical NF-κB can work in conjunction with IRF-3 as well as other enhancer components to maximize the expression of IFN-β gene.^124,125^ Abe et al. observed a 50% decrease in IFN-β production in primary mouse embryonic fibroblast cells when canonical NF-κB expression was partially silenced via RNA interference (RNAi).^119^ Similarly, canonical NF-κB and IRF3 are essential to induce type I IFN in DCs stimulated by irradiated tumor cell after IR.^126^ This is also consistent with another research which proved impaired canonical NF-κB pathway may recede IR-induced antitumor immunity.^126^ In contrast, it is reported that the release of IFN induced by IR may be inhibited by the non-canonical pathway in DC cells activated by STING pathway. Investigators demonstrated that cancer cells exposed to IR may promote the activation of the non-canonical NF-κB pathway in DCs through the process we discussed above. Taken together, the canonical and non-canonical NF-κB pathways may play the opposite role in regulating the type I IFN expression induced by the cGAS-STING signaling pathway in the context of RT.

The delivery of cGAS between cancer cells and non-cancer cells has been demonstrated. Like other IR-generated DAMPs, DNA derived from cancer cells can be recognized by DCs to exert immune response which is similar to Type I IFNs.^127^ For instance, it is reported that tumor-derived exosomes containing DNA can be transferred to DCs from tumor cells exposed to IR which leads to an upregulation of co-stimulatory molecules and STING-dependent activation of Type I IFN.^128^ In addition to DNA, it is reported that cytoplasmic tumor-derived cGAMP as the secondary messenger can diffuse to adjacent cells via gap junctions.^129^ The intercellular signaling may aid in the systemic immune response of RT.

### Effects of IR on stromal cells

Cancer-associated-fibroblasts (CAFs) are a heterogeneous population that make up the majority of stromal cells in many tumors. CAFs are relatively resistant to IR which stay alive in tissue culture when they are expose to ablative doses of radiation (18 Gy). Of note, following radiation, CAFs have persistent DNA damage and become senescent.^130^ DDR may activate downstream signaling and result in changes in molecules secreted by CAFs. Moreover, IR can recruit CAFs and myofibroblasts in the tumor microenvironment undergo to phenotypic transformation to CAFs.^131^ In particular, the expression of some angiogenic molecules is downregulation while the expression of fibroblast growth factor (FGF), and macrophage migratory inhibitory factor (MIF) is upregulation when CAFs are exposed to radiation.^132^ In addition, CAFs can promote the conversion to radioresistance cancer stem cells through the release of transforming growth factor-β (TGF-β).^133^ CAFs are known as immunosuppressors in tumor microenvironment and generally, CAFs promote tumor progression.^134^ However, it is reported that irradiated CAFs may change protumorigenic features which may lead to reduction of tumor engraftment and angiogenesis in vivo models.^135^. Of note, both irradiated CAFs and intact CAFs show the ability to mediate immunosuppression by reducing human T cells.^136^ More investigations and exploration are needed to characterize the specific immunosuppressive role of irradiated CAFs in this context.

### Immunomodulatory effect on various immune cells

**Fig. 2:**
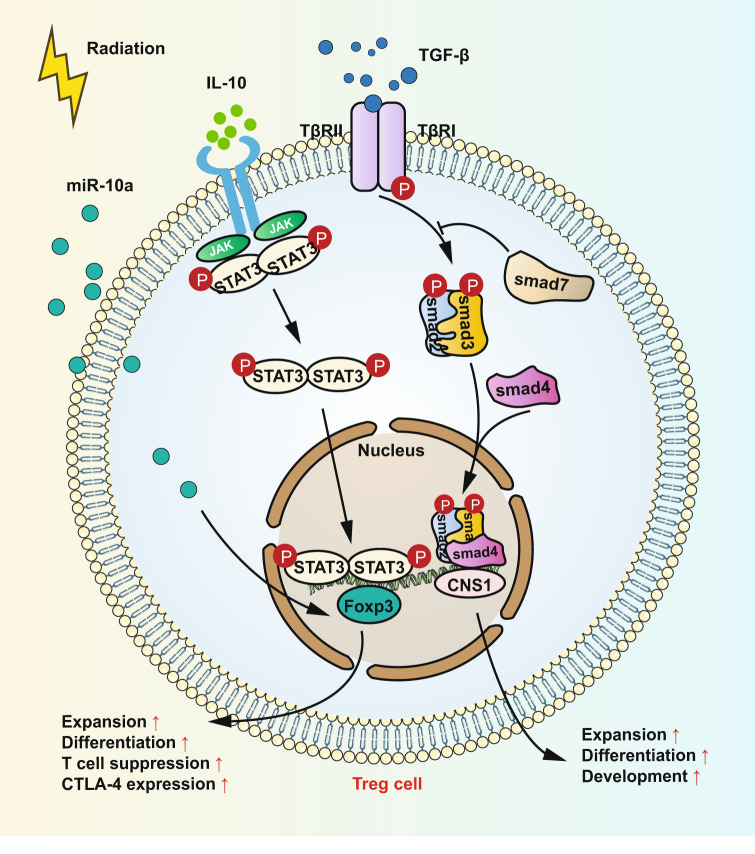
Immunomodulatory effect of radiation on Treg cells. Radiation promotes the conversion from CD4^+^ T cells to Treg cells and enhances Treg function through IL-10R-mediated STAT3 signaling pathway. Radiation also increases the secretion of IL-10 which can bind to IL-10 receptors. This binding activates JAKs and activated JAKs, such as JAK1 and JAK2, phosphorylate STAT3 at Tyr705, resulting in translocation of activated STAT3 dimers to the nucleus. STAT3, as a cotranscription factor with FOXP3, promotes expansion, differentiation, and T cell suppression and increases CTLA-4 expression of Treg cells. In addition, miR-10a induced by radiation can enhance the expression level of FOXP3 and promote the differentiation of Treg from naive CD4^+^ T cell. Radiation increases the level of TGF-β in the TME greatly and TGF-β recognizes and binds TGFβRII, which then phosphorylates TGFβRI. TGF-β activates Smad2 and Smad3 and promotes the formation of a heterotrimer with Smad4. Smads are recruited to the CNS1 region which has been identified at the Foxp3 gene locus. The CNS1 promotes generation, expansion, differentiation, and development of Treg cells. Parts of this figure were drawn with aid of Servier Medical Art (http://www.servier.com), licensed under a Creative Commons Attribution 3.0 Unported License. Parts of this figure were drawn with aid of Servier Medical Art (http://www.servier.com), licensed under a Creative Commons Attribution 3.0 Unported License

Treg cells are a type of CD4^+^ lymphocyte with the expression of the transcription factor forkhead box P3 (FOXP3); the existence of Treg cells has important implications for the maintenance of immune homeostasis and self‐tolerance.^137–141^ In cancer, Treg cells exert “unproductive” immunosuppression leading to unwanted immunosuppressive effects or even promoting disease progression.^140^ There are numerous studies indicating that Treg cell numbers in the tumor microenvironment increase significantly when tumor cells receive various types of radiotherapy.^142–144^ And elevated levels of Treg cells are related to a suppressive tumor microenvironment, the resistance of immunotherapy, and poor prognosis.^145–149^ Although IR leading to Treg cells infiltration is a generally acknowledged fact, the underlying mechanism is still not revealed. Muroyama et al.^143^ observed that the immunosuppressive function of Treg in the tumor microenvironment is potentiated by RT. Furthermore, mechanisms of Treg modulation by radiation are elucidated by Oweida and his colleagues in an orthotopic mouse model of head and neck cancer (HNC).^150^ They demonstrated that signal transducer and activator of transcription 3 (STAT3), as a key molecule to regulate FOXP3,^151,152^ promotes the conversion induced by radiation from CD4^+^ T cells to Treg cells and STAT3 inhibition in combination with radiation changes tumor microenvironment dramatically in the aspect of decreasing Treg cells, MDSCs and M2 macrophages along with enhancing effector T cells and M1 macrophages. This result also corroborates previous studies implicating that STAT3 signaling may play a crucial role in enhanced Treg function and conversion.^153^ The underlying mechanism is IL-10R-mediated STAT3 signaling pathway. IL-10 binds to its receptor and activates STAT3 signaling which then promotes the proliferation of Foxp3(+) iTregs with increased level of CTLA-4 and potentiated immunosuppressive function with the help of TGF-β. Similarly, this process may occur when using blockade PD-1 in vitro. It is reported that PD-1 blockade also increases the release of IL-10 by T cells, leading to higher Tregs proliferation.^154^ However, Woods found that Treg suppression is reduced in vitro by PD-1/PDL1 blockade and enhanced by STAT3 inhibition.^155^ And in accordance with their research, previous studies proved that STAT3 expression in Tregs has a close relation to decreased suppressive function.^156^ These contrary data show that more investigations are needed to explore the role of STAT3 expression for patients with tumor in response to different treatments but STAT3 signaling and IL-10 production may play a critical role in the proliferation and function of Treg. Of note, the type I IFN production and signaling pathway and NF-κB pathway involve in the production of IL-10.^157^ As we discussed above, cGAS-STING induced by IR activates type I IFN and NF-κB signaling pathway which then lead to IL-10 production. Specifically, IL-10 binds to its receptor and activates the JAK–STAT3 signaling which then exerts the biological effect.^158,159^ Moreover, increased TGF-β production induced by RT may favor Treg accumulation in the tumors.^160^ ROS induced by RT may promote the conformational change of the latency-associated peptide/TGFβ complex which leads to the secretion of TGF-β when tumor cells receive RT.^161^ Many studies have confirmed the critical role of TGF-β for iTreg development and IR greatly increases the level of TGF-β in the tumor microenvironment.^162–166^ In Treg cells, TGF-β activates Smad2 and Smad3 through TGF-β receptors and promotes the formation of a heterotrimer with Smad4. The heterotrimer translocates into the nucleus and binds to the conserved non-coding sequence 1 (CNS1) which is located in the Foxp3 gene locus as an intronic enhancer.^167^ Importantly, there are two consecutive Smad-binding sites CNS1 it has been demonstrated that CNS1 plays an important role in iTreg/pTreg generation.^168^ In addition, miR10a/10b expression has been demonstrated to be associated with TGF-β expression and it is reported that TGF-β induces miR10 expression in Treg cells.^169^ And miR-10a can increase the expression level of FOXP3 and facilitate the differentiation of Treg from naive CD4^+^ T cell.^170^ IR not only increases the amount of Treg cells, but also enhances the radioresistant capacity of Treg. It was shown that Tregs surviving after RT had an upregulated Akt expression, which rendered them more resistant to RT-induced apoptosis.^171,172^ In addition to the increasing count of Treg cells, IR can modulate the phenotype and function of human Treg cells. Beauford and colleagues found that IR can attenuate the function of Treg to suppress the proliferation of CD8^+^ T cells through downregulating Foxp3 expression and modulating the expression of Treg signature molecules, for example, increasing the expression of lymphocyte activation gene 3 (LAG-3) and decreasing the expression of CD25 and CTLA-4.^142^ Kumagai et al.^147^ found Treg cells has indicative significance for the prediction of PD-1 therapeutic effect. They confirmed that the frequency of PD-1^+^CD8^+^ T cells has a close relation to that of PD-1^+^ Treg cells in the tumor microenvironment and this ratio may be a better predictor for PD-1 therapeutic effect. The signaling pathways of immunomodulatory effect on Treg cells are summarized in Fig. 2.

**Fig. 3:**
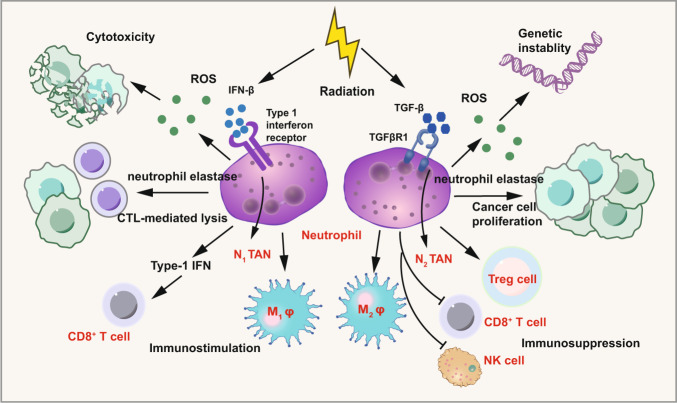
Immunomodulatory effect of radiation on TANs. Radiation shows complexity regarding the immunomodulatory effect on TANs. On the one hand, radiation may induce TANs to exhibit the antitumor characteristic (N1) by IFN-β. N1 phenotype induces tumor cells cytotoxicity/apoptosis through ROS and activates CD8^+^ T cells and M1 macrophage. On the other hand, radiation may induce TANs to exhibit the pro-tumor characteristic through TGF-β. N2 phenotype promotes genetic instability by ROS, cancer proliferation, and immunosuppression effect by inhibiting CD8^+^ T cells and NK cells and enhancing Treg cells. Parts of this figure were drawn with aid of Servier Medical Art (http://www.servier.com), licensed under a Creative Commons Attribution 3.0 Unported License

According to functional differences, tumor-associated neutrophils (TANs) can be divided into two subtypes: antitumorigenic or protumorigenic neutrophils, termed N1 and N2, respectively.^173–177^ TANs can be a component of tumor-promoting inflammation by promoting angiogenesis, immunosuppression, remodeling of the extracellular matrix, and metastasis. The interaction between neutrophil polarization and tumor microenvironment has been well summarized but there are few studies focusing on the relationship between TANs polarization and IR.^178–184^ Generally, TGF-β, an immunosuppressive cytokine overexpressed by tumor cells, polarizes neutrophils to a protumorigenic phenotype (N2) and inhibits N1 phenotypic polarization. On the contrary, IFN-β polarizes neutrophils to an antitumorigenic phenotype (N1) while inhibits N2 polarization.^185^ The function of N1 involves in promoting tumor cell cytotoxicity/apoptosis, strengthening the antibody-dependent cellular cytotoxicity (ADCC), and activating T cells.^186–189^ N2 phenotype shows the protumorigenic characteristics including promoting the tumor growth, stemness, angiogenesis, invasion, and suppressing immunity.^190,191^ However, to date our knowledge on the role of N1 and N2 in RT responses remains limited and contradictory. Similar to DCs, IR activates neutrophils through toll-like receptors (TLR)-dependent mechanisms.^192^ Neutrophils recognize DAMPs which are TLRs agonists after RT and the ligation of TLRs enhances the immune responses directed against tumor-associated antigens (TAAs). On the one hand, RT may induce TANs to exhibit the pro-tumor characteristic, since neutrophils may promote resistance to RT.^193–195^ Wisdom and his colleagues found that elevated neutrophil levels have a close relation to poor outcome of patients with cervical cancer after chemoradiation. Similarly, others have found that genetic depletion of neutrophils improves RT response in a genetically engineered mouse model of sarcoma. Notably, evidence has verified that anti-Ly6G antibody-mediated neutrophil depletion may lead to an improvement of the RT efficacy.^196,197^ These data suggest that neutrophils play a crucial role in the tumor microenvironment following RT which may promotes the conversion of N1 to N2. Thus, targeting neutrophils and their immunosuppressive effector molecules, TGFβ and nicotinamide phosphoribosyltransferase (NAMPT) might be crucial to reversing immunosuppression.^198^ Besides the pro-tumor characteristics, TANs also exhibit antitumor characteristics by inhibiting the growth of tumor cells, and interacting with other immune cells which is induced by RT. Takeshima et al.^199^ observed that RT may lead to sterile inflammation by infiltrating CD11b^+^Gr-1^high+^ neutrophils into the tumors rapidly and transiently and sterile inflammation eventually activated tumor-specific cytotoxic T cells which may result in tumor regression. Liu et al.^200^ proved that RT can promote the recruitment of neutrophils and that the radiosensitivity can be improved by neutrophils which inhibit the process of epithelial-mesenchymal transition via the ROS-mediated PI3K/Akt/Snail signaling pathway. As we discussed above, TGF-β polarizes neutrophils to a protumorigenic phenotype (N2) and inhibits N1 phenotype. However, RT has also been demonstrated as an inhibitor of the TGF-β pathway, thereby stimulating the antitumor-N1 neutrophil phenotype polarization.^179^ Once more, the contradictory roles of neutrophils in RT responses may reflect differences in tumor phenotypes (Fig. 3).

**Fig. 4:**
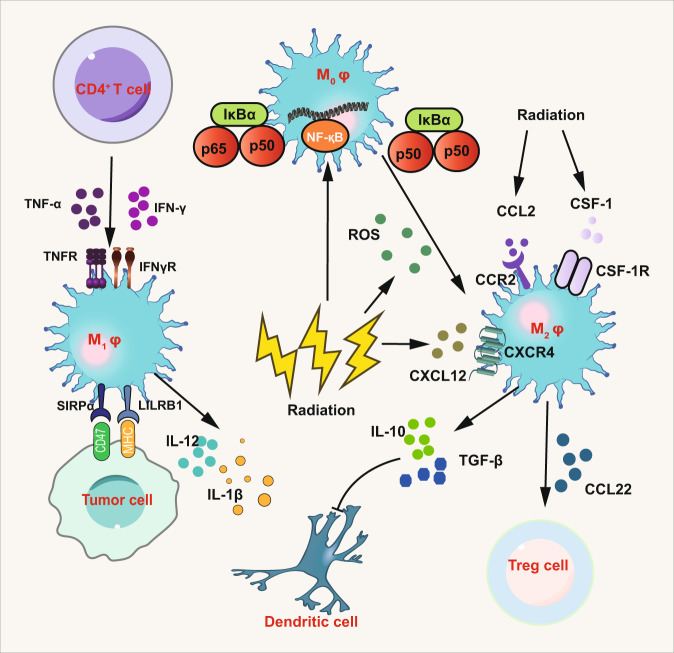
Immunomodulatory effect of radiation on TAMs. Under the effect of p50–p50 NFκB homodimer induced by radiation, M2 macrophages acquired their phenotype. Meanwhile, increased ROS caused by radiation also promotes the polarization to M2 macrophage. The activation of p50–p50 dimer promotes the conversion towards M2 phenotype, leading to the secretion of IL-10 and TGF-β which inhibit DCs. And CCL22 secreted by M2 macrophage also recruits Treg cells to exert immunosuppressive function. Radiation elicits a high recruitment of TAMs through CCL2/CCR2 and CSF1/CSF1R pathways. And it can also recruit TAMs to infiltrate tumor sites, especially hypoxia sites through SDF-1/CXCR4-dependent signaling pathways. Moreover, M1 macrophage ban be activated by CD4^+^ cells through TNF and IFN-γ and then kill tumor cells via phagocytosis which plays a crucial role in abscopal effect. Parts of this figure were drawn with aid of Servier Medical Art (http://www.servier.com), licensed under a Creative Commons Attribution 3.0 Unported License

**Fig. 5:**
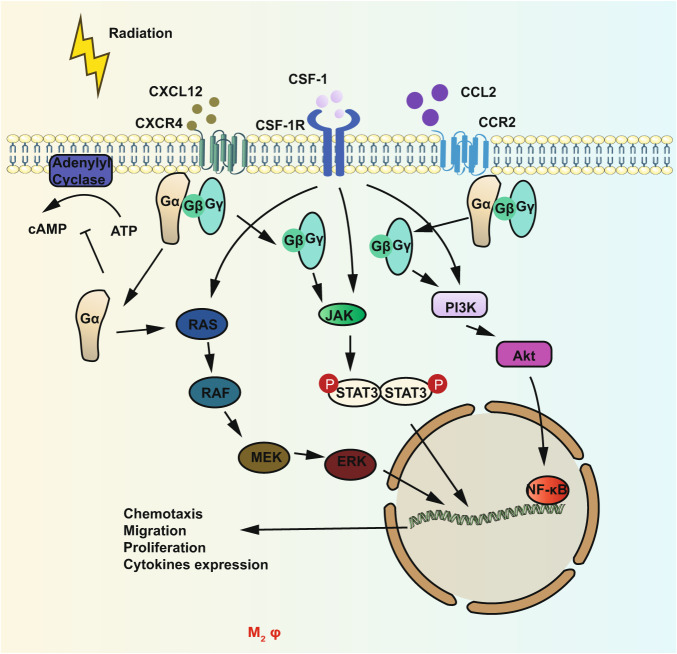
Signaling pathways of radiation on TAMs. Radiation increases the level of CXCL12 which then binds to CXCR4, a kind of G-protein-coupled receptor (GPCR). Similarly, CCL2 and its receptor, CCR2 are also activated by radiation. Next, the receptor undergoes a second conformational change that activates the intracellular trimeric G protein by the dissociation of Gα subunit from the Gβ/Gγ dimer. Then, the phosphatidylinositide 3-kinases (PI3Ks) can be activated by both Gβ/γ and Gα subunits. PI3Ks regulate gene transcription, migration, and adhesion of TAMs by the phosphorylation of AKT and of several focal adhesion components. In addition, Gα subunit also activates the Ras and Rac/Rho pathways, leading to the phosphorylation of ERK. Activated ERK can phosphorylate and regulate other cellular proteins, as well as translocate into the nucleus and phosphorylate and regulate transcription factors, leading to migration, proliferation, and cytokines expression of TAMs. Gβ/Gγ dimer also activates JAK/STAT signaling pathway to promote changes in cell morphology leading to chemotactic responses. Of note, CSF-1/CSF-1R activates all the three signaling pathways we discussed above. Parts of this figure were drawn with aid of Servier Medical Art (http://www.servier.com), licensed under a Creative Commons Attribution 3.0 Unported License

Similar to TANs, tumor-associated macrophages (TAMs) can be classified into two categories, a tumor-cell-killing phenotype of macrophage called M1, and the other being a tumor-promoting phenotype called M2,^201–208^ although in reality there is a spectrum-like level of activation that cannot be simply classified as M1 and M2.^209^ Brown et al.^210^ reviewed a series of evidence that had explored the significance of the increasing TAMs in the tumor microenvironment after IR in preclinical studies and clinical trials of newly diagnosed glioblastoma patients. Akkari et al.^211^ reported the dynamic changes in glioma macrophage populations after RT and altered expression of several genes and proteins in recurrent human glioblastoma. The mechanisms for radiation-induced alteration of TAMs are unclear. Generally, IR elicits a high recruitment of TAMs through chemokine (C-C Motif) ligand 2 (CCL2) and colony-stimulating factor 1 (CSF1) pathways, and IR can also cause oxygen deprivation and upregulate hypoxia-inducible factor (HIF) which recruits TAMs to infiltrate tumor sites, especially hypoxia sites through stromal cell-derived factor 1 (SDF-1)/C-X-C chemokine receptor type 4 (CXCR4)-dependent signaling pathways (Fig. 4).^212–214^ These recruited macrophages adopted an M2-like pro-tumoral phenotype with enhanced pro-survival and pro-angiogenic activities, often leading to tumor recurrence and treatment failure.^215^ In addition to the slight changes of IR to the viability of macrophages, it is reported that IR can also modify the macrophage phenotype. Genard et al.^216^ systematically summarized the dose-dependent effects of IR to polarize macrophages. Generally, numerous studies have implicated that TAMs may polarize to M2 phenotype in the context of low-dose IR while high-dose IR may promote the polarization to M1 in vitro.^217,218^ Thus, IR promotes not only M1 activation of TAMs but also facilitates M2 activation. Although Meng et al.^219^ have investigated that a large single dose (20 Gy) or at 2 Gy in 10 fractions (10 × 2 Gy) promotes the conversion of immunosuppressive TAMs, but some studies revealed a contrary effect of short-course or low-dose RT on TAMs. A study shows low-dose radiation programs macrophage differentiation to an inducible nitric oxide synthase (iNOS)^+^/M1 phenotype and these iNOS^+^/M1 macrophages can orchestrate effective T cell recruitment and kill tumor cells through iNOS.^220^ Moreover, total body low-dose radiation also promotes the polarization of TAMs towards an iNOS+/M1 phenotype.^221^ Another study shows short-course neoadjuvant RT reprograms macrophages towards an M1 phenotype in rectal cancer patients.^222^ These conflicting results may reflect the complexity and plasticity of TAMs and more investigations into how RT affects macrophage polarization are needed. The mechanisms about the effects of radiation dose on the polarization of TAMs remain unclear but there are several possible interpretations including ROS, NF-κB signaling, and MAPK phosphorylation (Fig. 5). One of the most critical mechanisms is the NF-κB balance. It is reported that p50–p50 NF-κB homodimer may promote the polarization towards M2 macrophages while p50–p65 NF-κB heterodimer favors the polarization towards M1 macrophages.^223^ Crittenden et al. demonstrated that M2 phenotype emerged in the context of high-dose irradiation (60 Gy) which activated p50–p50 dimer and promoted the secretion of IL-10.^224^ Of note, ROS is known as a secondary messenger which leads to secretion of a series of proinflammatory cytokines. However, it has been proved for the M2 activation.^225^ Tabraue et al.^226^ demonstrated the role of liver X receptor (LXR) nuclear receptors on radiation-induced polarization of macrophages in LXR double knock-out mice models. Moreover, the regulatory function of CAFs on macrophages after RT has been proved.^227^ The interaction between CAFs and M1-macrophages showed that CAFs can inhibit the function of M1-macrophages by reducing M1-surface markers expression in the context of RT. And tumor-vasculature development via endothelial-to-mesenchymal transition after RT may play a crucial role in macrophage polarization.^228^

**Fig. 6:**
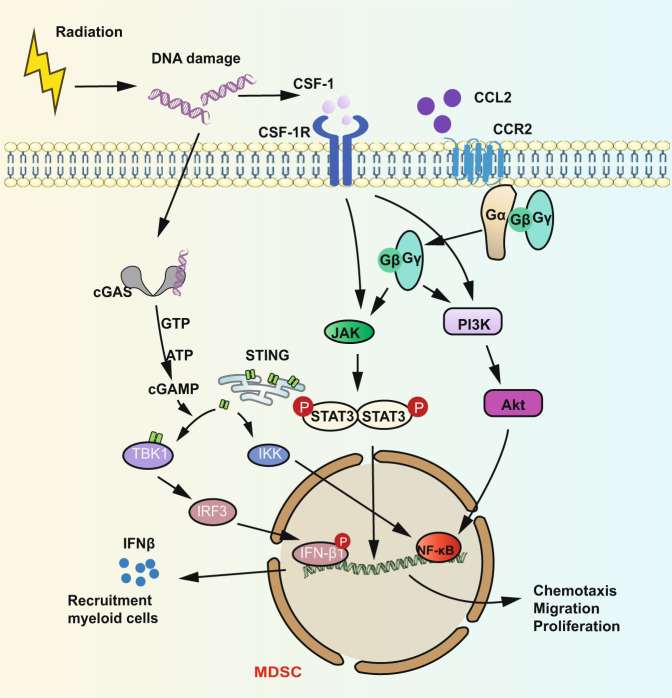
Immunomodulatory effect of radiation on MDSCs. DNA damage resulted from radiation may activate two signaling pathways in MDSCs: cGAS-STING and JAK/STAT signaling pathways. Similar to signaling pathways in TAMs, JAK/STAT signaling and PI3K/AKT signaling pathway are activated in MDSCs caused by CSF-1/CSF-1R and CCL2/CCR2. Upon DNA damage, all of these damaged DNA including cytoplasmic DNA and mtDNA induced by IR can be recognized by cGAS, which then oligomerizes with DNA in the form of a 2:2 complex.^114–116^ After binding to DNA, cGAS then exerts a catalytic role to promote the synthesis of the second messenger 2′3′-cyclic GMP–AMP (cGAMP). Binding of 2′3′-cGAMP stimulates STING and promotes the translocation to the Golgi which acivates TANK-binding kinase 1 (TBK1). TBK1 phosphorylates STING and promote the interferon regulatory factor 3 (IRF3) to translocate to the nucleus which triggers the expression of IFN-β gene. Parts of this figure were drawn with aid of Servier Medical Art (http://www.servier.com), licensed under a Creative Commons Attribution 3.0 Unported License

MDSCs are the “queen bee” of the tumor microenvironment that protect the tumors and contribute to tumor progression.^229–235^ In normal conditions, bone marrow-derived myeloid cells can differentiate into cells of the innate immune system including macrophages, dendritic cells, and granulocytes. But in a pathological condition, myeloid cell precursors may be proliferated and differentiated into MDSCs that migrate to the tumor microenvironment and develop into TAMs which are induced by inflammatory cytokines, for instance, soluble tumor necrosis factor (sTNF). MDSCs have a close relation with TAMs and TANs in the tumor microenvironment. Polymorphonuclear (PMN)-MDSCs seem to develop into N2 TANs while monocytic (M)-MDSCs seem to proliferate and develop into TAMs under chemotaxis of several factors, such as CCL2, CXCLs, and S100A8/A9, etc.^236^ And signals underlying the development, differentiation, and recruitment of MDSCs are complex and involve granulocyte-macrophage colony-stimulating factor (GM-CSF), granulocyte (G)-CSF, macrophage (M)-CSF and vascular endothelial growth factor (VEGF); cytokines such as IL-4, IL-6, IL-10, IL-1β, interferon IFN-γ, TGF-β, and prostaglandin E2 (PGE2); chemokines such as CCL2, CXCL5, and CXCL12. MDSCs can exert the immunosuppressive effect through releasing TGFβ, and IL-10, and inducing Treg cells.^35,237–243^ In almost all patients with tumors, MDSCs seem to be the major factor to protect the tumors and contribute to tumor progression. Zeng et al. found that caspase-1 from human MDSCs may lead to T cell-independent tumor proliferation through inactivating T cells and NK cells.^244^ Moreover, MDSCs also have the function to facilitate the expansion and activation of Treg cells. A study showed that monocytic MDSCs which is isolated from transplant patients can suppress the proliferation of CD4^+^ T cells and promote the expansion of Treg cells.^245,246^ It is reported that a significant increase of MDSCs in some organs or tissues of the human body such as spleen, lung, lymph nodes, and peripheral blood was observed when primary tumor sites received RT.^247^ However, other groups reported that there was a dramatical decrease of MDSCs 7–14 days after the murine colon tumors received a single high dose of RT.^196,248^ These contrary data might be related to the various tumor models and difference of radiation doses, fractionation, irradiated sites as well as the timings of the analyses. CSF1/CSF1R signaling pathway plays a key role in the infiltration of MDSCs into tumors in the context of RT. The blockade of this signaling pathway can improve tumor recurrence post local RT. Xu and colleagues proved the importance of CSF1/CSF1R signaling in the recruitment of MDSCs which can limit the efficacy of RT.^247^ The further studies revealed that the accumulation and translocation of the DNA damage-induced kinase ABL1 may enhance the transcription of CSF1 gene which lead to the significant release of CSF1 in prostate post-RT. And Liang et al. found a mechanism by which extrinsic resistance develops after local ablative radiation that relies on the immunosuppressive action of STING of MDSCs.^249^ The STING/type I interferon pathway enhances suppressive inflammation in tumors by recruiting myeloid cells in part via the CCR2 pathway (Fig. 6). Intriguingly, it is reported that activation of STING pathway can reprogram MDSCs into immunostimulatory cells.^250^ However, majority of studies showed that STING signaling pathway in the tumor microenvironment can inhibit the recruitment, differentiation, and function of MDSCs.^100^ Cheng et al. observed that the activation of STING signaling pathway by cGAMP may enhance the secretion of IFN-γ but decrease the number of MDSCs in vivo.^251^ Therefore, these studies suggest the suppression function of IR on MDSCs. In summary, cGAS/STING signaling is vital in the context of RT and its complex effect on MDSCs need to be exploited. In pancreatic cancer, the potential mechanism of intensive immunosuppression of MDSCs after RT is by increasing lactate secretion induced by Warburg effect.^252^ And the essential factor to mediate lactate-regulated activation of MDSCs is HIF-1α which activates HIF-1α/STAT3 pathway. Another study showed that silencing information regulator 2 related enzyme 1 (SIRT1) can deregulate function and differentiation of MDSCs through orchestrating HIF-1α-dependent glycolysis.^253^ Of note, IR generally induces the recruitment and infiltration of MDSCs to yield immunosuppressive effect on the immune system (as discussed above).

**Fig. 7:**
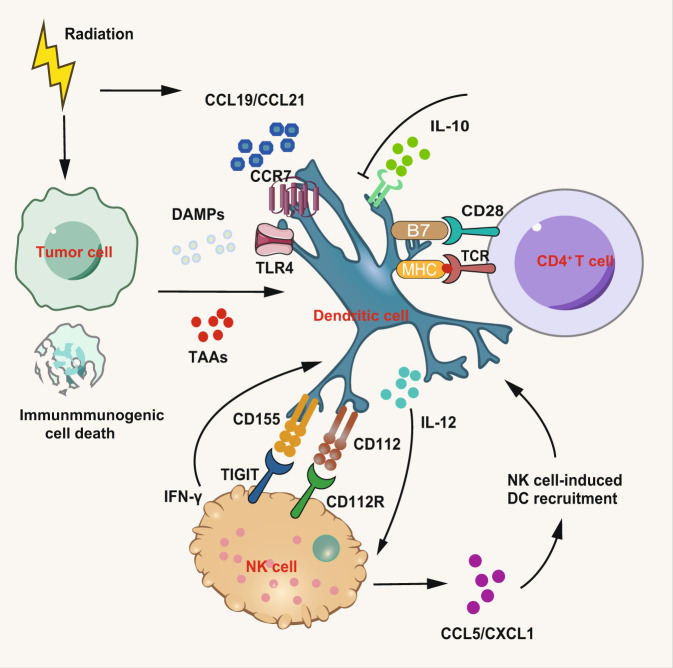
Immunomodulatory effect of radiation on DCs. Radiation on DCs significantly increases the expression of chemokines CCL19 and CCL21 which then bind to CCR7 and mediate migration of DCs. In addition, immunogenic cell death caused by radiation may release large amounts of antigens and DAMPs including HMGB1, ATP, calreticulin, heat shock proteins, and other cellular factors which bind specific pattern-recognition receptors on the dendritic cell, including Toll-like receptors, RIG1-like receptors, and NOD-like receptors. And radiation also results in the release of TAAs which promotes DCs activation, migration, and proliferation of T cells. Parts of this figure were drawn with aid of Servier Medical Art (http://www.servier.com), licensed under a Creative Commons Attribution 3.0 Unported License

Dendritic cells are the most potent antigen-presenting cells for their specialized dendritic morphology, as the essential component to link innate and adaptive immunity.^254–260^ The most common contributors to activate DCs are pathogen-associated molecular pattern molecules (“danger signals”) through the respective receptors. But in the case of RT, DCs are mainly activated by DAMPs including HMGB1 and calreticulin. Gupta and colleagues observed that after tumor RT, there is an upregulation of two co-stimulatory molecules, CD70 and CD86 on DCs.^85^ On the one hand, the initial factors are increased by IR and the mechanisms that IR can upregulate the expression of class I MHC have been well established.^84,261–263^ And immunogenic cell death caused by radiation may release large amounts of antigens and DAMPs including HMGB1, ATP, calreticulin, heat shock proteins, etc.^264^ These molecules can further activate DCs through binding to the receptors which comprise TLRs, retinoic acid-inducible gene 1 (RIG1)-like receptors, and nucleotide-binding and oligomerization domain (NOD)-like receptors. For instance, calreticulin increased by RT acts as a pro-phagocytosis eat-me signal in opposition to CD47.^265^ Release of HMGB1 from tumor cells, via TLR4 activation, promotes antigen presentation by DCs.^266^ In addition, IR can strengthen tumor cross-presentation of DCs which may promote the activation and proliferation of T cells.^267,268^ The release of DAMPs and TAAs promotes DCs to migrate towards lymph nodes and further results in the emergence of systemic antitumor immune responses. On the other hand, inflammatory cytokines modified by RT may augment the function of DCs and potentially exert an immunostimulatory response to radiation.^34^ Moreover, new mechanisms have been proved by researchers (Fig. 7). Yu and colleagues found that in vitro when DCs are exposed to 0.2 Gy radiation, there is an increase of migration mediated by CCR7 and IL-12 production induced by the ATM/NF-κB pathway.^269^ For high dose of radiation, Zhou found that it may promote DCs homing and T cells priming through facilitating the ROS-induced cytoskeletal reorganization.^86^ In addition to the positive immune effect of radiation to DCs, DCs may also play a crucial role in radiation-induced immunosuppression. Liu et al. observed a decrease of CD8^+^ DC both in patients and mice after IR which causes shift from Th1 to Th2 immunity and this process might be mediated by Fms-like tyrosine kinase 3 ligand (FLT3 ligand), a CD8^+^DC-inducing cytokine.^270^

Generally speaking, B cells and T cells are sensitive to IR, so IR damage to these lymphocytes which may lead to adverse events of RT. Within the lymphocyte population, B cells are the most sensitive to IR. Compared with B cells, T cell subsets show different sensitivity to IR. Thus, the sensitivity to IR for T cells has been explored in a large body of studies but there are a large of conflicting results.^271^ Arina et al.^272^ found that RT can reprogram a huge proportion of T cells which show increased motility and increased secretion of IFN-γ. In addition, T cells within the tumor may be more resistant to IR compared with naive and circulating T cells. Generally, CD4^+^ T cells are considered more radioresistant than CD8^+^ T cells, with Treg cells even more resistant to RT.^271,273,274^ Except the direct damage to T cells of IR, it also activates T cell through increasing major histocompatibility class I (MHC-I), DAMPs, and TAAs and enhances T-cell infiltration. Moreover, these molecules as well as increased proinflammatory cytokines augment the prime of T cells to exert an antitumor response with the migration of DCs towards adjacent lymph nodes^95^. It has been proven that IL-1β, TGF-β, fibroblast growth factor (FGF), and TNF, as well as NACHT, LRR, and PYD domains-containing protein 3 (NALP3)-inflammasome activation and signaling are key components to mediate the response to IR.^275–278^ Recently, Lin and colleagues found that radiation-induced small extracellular vesicles may play a role as efficient carriers containing TAAs and DAMPs in promoting tumor antigen release and triggering antitumor immunity.^83^ Moreover, a study has proved that radiation can promote tumor cells to release more MHC-I molecules in murine tumor models, which facilitates cross-presentation and T-cell priming.^279^ In fact, the high release of TAAs and MHC-I has been already reported in human carcinoma cell lines when researchers use sublethal irradiation on human tumor cells.^89^ This result just accords with the role of low-dose RT in patients who receive SBRT combined with IO, which better facilitates the abscopal effect and increases the occurrence rate in patients with metastatic disease in iRT.^280^ Moreover, RT induces the production and release of cytokines. The most important mechanism is to activate the cGAS-STING and NF-κB signaling leading to an increase of cytokines.^128,281,282^ Some investigations show that IR promotes the secretion of CXCL16 in tumor cells which can be combined with Th1 cells and CXCR6 on activated CD8^+^ T cells to increase the infiltration of local immune cells.^283,284^ On the other hand, IR may promote the migration of T cells towards irradiated tumor sites resulting in a positive immunological outcome. In this process, the intercellular adhesion of molecule-1 (ICAM-1) may promote leukocytes migration to the endothelial cells, and reprogram an inflammatory tumor microenvironment. This is enhanced by IR and this leads to more T-cell infiltration into tumor tissues.^285–287^ It is reported that the density of CD3^+^ and CD8^+^ lymphocytes in tumor site is associated with disease-free and overall survival in patients treated with chemoradiotherapy for rectal cancer.^87^ More recently, Dovedi et al.^288^ found that low-dose RT combined with PD-1 blockade can promote the migration of T-cells to primary treated sites and augment tumor regression. Thus, RT enhances the secretion of cytokines which may promote T-cell infiltration and augmenting T-cell priming.

**Fig. 8:**
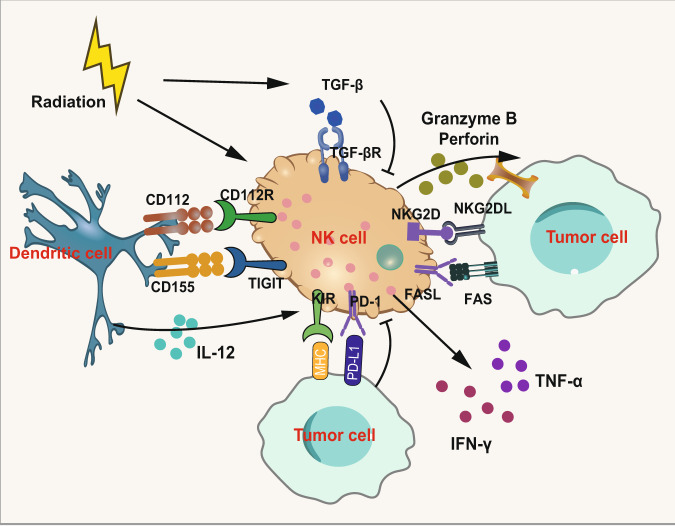
Immunomodulatory effect of radiation on NK cells. On the one hand, radiation inhibits NK cells through increasing TGF-β. Moreover, the exposure of MHC-I molecules on the surface of tumor cells caused by radiation also inactivates NK cells through KIR. In addition, radiation induces the upregulation of activating receptors NKG2D and NKG2D ligands (NKG2DLs) to enhance the function of NK cells via granzyme B and perforin, FAS and FAS ligand and ADCC. Parts of this figure were drawn with aid of Servier Medical Art (http://www.servier.com), licensed under a Creative Commons Attribution 3.0 Unported License

NK cells are cytotoxic lymphocytes in the innate immune system to kill cancerous cells. The two most well-characterized subsets of NK cells are the CD56^bright^CD16^−^ and CD56^dim^CD16^+^ populations.^289–292^ In the subsets of NK cells, the CD56^dim^ NK cell population is the major contributor to kill infected and malignant cells. The main mechanisms are granzyme B and perforin, FAS and FAS ligand and ADCC. For the direct effect on tumor cells and cellular cross-talk, NK cell-based immunotherapies have been explored well.^293–300^ The activation of NK cells depends on the balance between activation and suppression signals.^301^ The activating receptors of NK cells such as NKG2D can promote the secretion of cytokines and enhance the cytotoxicity and an increasing expression of NKG2D ligands was observed by investigators in several human cancer cell lines after IR.^301,302^ The increasing expression of NKG2D ligands (NKG2DLs) induced by radiation may further activate the NK pathway and NKG2D-based CAR T cells combined with RT can exert synergistic efficacy in glioblastoma (Fig. 8).^303^ Meanwhile, inhibitory signaling pathways including PD-1, NKG2A and killer immunoglobulin-like receptors (KIRs) play a key role in function of NK cells so ICIs can attenuate suppression of NK cell through binding to these inhibitory signaling pathways.^304^ Furthermore, other molecules such as MHC-I induced by RT may also inhibit the function of NK cells. In addition to increasing NKG2D ligands, IR enhances NK cell homing and cytotoxicity in canine models of sarcoma.^305^ Researchers observed significantly increased NK cells homing to tumors in vivo and increased activation of circulating NK cells after RT which yield tumor regression and abscopal responses. This has also been shown in a human triple-negative breast cancer xenograft model.^306^

### IR reprograms the tumor microenvironment

IR not only reprograms the tumor microenvironment from “cold” to “hot”, but also exerts immunoinhibitory effects in the tumor microenvironment.^307^ In the above, the authors have discussed and summarized the effects of IR on cancer cells, stromal cells, and immune cells which lay a basis for reprogramming the tumor microenvironment. Along with this process, activation of intrinsic signaling pathways especially cGAS-STING signaling, secretion of chemokines and cytokines (Table 1), formation of hypoxia condition, and activation of the classical and alternative complement system play a crucial role in reprogramming tumor microenvironment induced by IR.^308–312^ The process of reprogramming the tumor microenvironment is suffused with complex and profound changes, which is not only limited to a change in quantity and form, but also full of various interactions and modulations. We have discussed this process in the above article. Besides, many reviews have given an unambiguous and detailed interpret of reprogrammed tumor microenvironment and have shown potential benefit of reprogrammed tumor microenvironment induced by RT to combine with IO.^123,146,313–318^

**Table 1:** Immunomodulatory factors and their functions in TME induced by radiation

Factors	Receptors	Function
*Chemokines*		
CXCL2	CXCR2	TAM infiltration and differentiation
CXCL12	CXCR4, CXCR7	TAM infiltration and differentiation
CCL2	CCR2	TAM infiltration and differentiation
		Monocyte recruitment
		MDSCs recruitment
CCL3, CCL5	CCR1, CCR4, CCR5/CCR5	TAM infiltration and differentiation
CCL7	CCR1, CCR2, CCR3, CCR5	DCs migration
CCL21	CCR7	DCs migration
CCL22	CCR4	Treg recruitment
CCL28	CCR3, CCR10	Treg recruitment
*Cytokines*		
TGF-β	TGFβRI, TGFβRII	M1 → M2 TAM polarization
		N1 → N2 TAN polarization
		Naive CD4^+^ T cell into Treg
		NK suppression
TNFα	TNFR	T cell proliferation
		T cell action
		M1 macrophage polarization
IL-1	IL-1R	MDSCs induction
		Macrophage recruitment
IL-2	IL-2R	T cell proliferation and effector function
IL-4	IL-4Rα	Macrophage recruitment
IL-10	IL-10R	Proinflammatory cytokines inhibition
		Antigen presentation inhibition
		Treg action
		Macrophage inhibition
IL-12	IL-12R	DCs migration
		T cell priming
		Upregulates MHC I
		Promotes CD8^+^ T cell-mediated cytotoxicity
IL-18	IL-18R	NK enhancement
IL-33	IL-33R	Activation of immune cells
IL-37	IL-18R	NK suppression
IFN-β	Type I interferon receptor	N2 → N1 TAN polarization
IFN-γ	Type II interferon receptor	Proinflammatory cytokine release
*Growth factors*		
VEGFA	VEGFR-1	Treg proliferation
		MDSC accumulation
		Myeloid cell differentiation
		T cell inhibition
		CTLA-4, PD-1 expression by CD8^+^ T cell
PGE2	Prostanoid E (EP) receptor	Proinflammatory cytokine release
CSF1	CSF1-R	TAM mobilization and proliferation
		M1 → M2 TAM polarization
		MDSCs recruitment
G-CSF	G-CSF receptor	Neutrophiles mobilization

### iRT enhcances abscopal effect

**Fig. 9:**
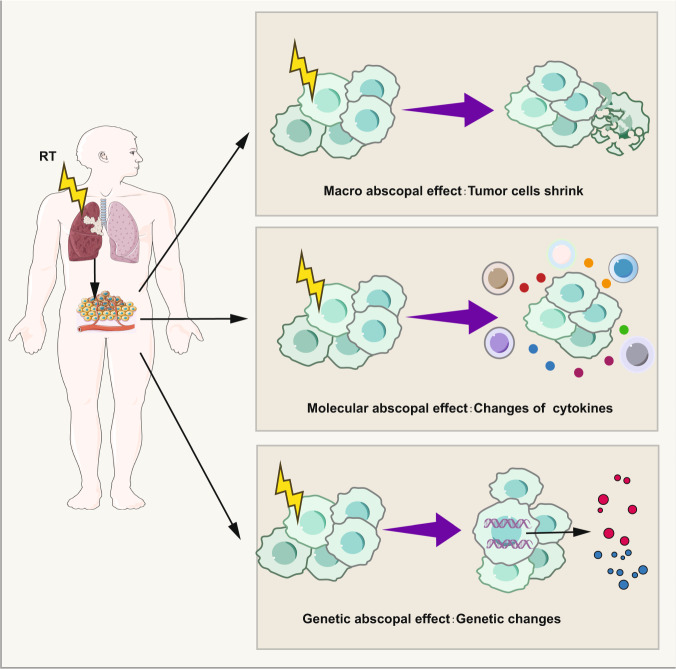
Macro, molecular, and genetic abscopal effect. Abscopal effect in the traditional sense refers to the tumor regression at distant non-irradiated sites which can be observed in clinic. However, when the primary tumor is irradiated, cytokines and immune cells at distant non-irradiated sites also changes due to the systemic immune response caused by IR. In addition, there are alterations of gene expression at distant non-irradiated sites. Parts of this figure were drawn with aid of Servier Medical Art (http://www.servier.com), licensed under a Creative Commons Attribution 3.0 Unported License

Traditionally, abscopal effect refers to an interesting phenomenon that local radiation may exert a systemic antitumor immune response and lead to the regression of non-irradiated distant tumors. Unfortunately, it is so rare for the occurrence rate of abscopal effect that it has no broad application value seemingly. Whereas, with the advent of immunotherapy, it seems that using radiotherapy combined with immunotherapy to enhance abscopal effect is available. A large number of studies about abscopal effect induced by iRT has come out.^21,48,319–326^ Nowadays, the criterion for determining whether abscopal effect has occurred is whether the tumor regression at distant non-irradiated sites can be observed. In view of the combination of RT with IO, the immune state of the whole human body and have already changed before tumor regression at distant non-irradiated sites (macroscopic abscopal effect). This force is not powerful enough to decrease tumor volume although molecules and gene expression of tumor cells or normal tissue cells nearby really do change. Thus, we propose a broad concept of abscopal effect from three aspects: macro level, molecular and genetic levels. From this point of view, the occurrence of abscopal effect is 100%, since most non-macro-level abscopal effects occur microscopically and in a subclinical manner (Fig. 9).

Heretofore, we have elaborated on immunomodulatory effect of IR acting on the primary irradiated sites. It is widely accepted that in situ vaccination induced by IR is the key mechanism transforming local effects into abscopal responses.^13,99^ Generally, the immunogenic death of tumor cells caused by IR releases plenty of neoantigens and DAMPs leading to an increasing of antigen presentation through DCs.^323^ With the undergoing maturation of DCs, activated cytotoxic T lymphocytes (CTLs) and NKs then recognize and attack both primary and abscopal tumor cells.^319,327^ Moreover, damaged nuclear DNA in the cytosol can be sensed by cGAS-STING pathway leading to IFN production along this pathway which has a crucial role in T-cell cross-priming to initiate an antitumor response.^328^ Similar to the above mentioned, the activated CTLs and NKs move to tumor sites via blood circulation and control tumor growth at both irradiated and no-irradiated sites.^320^ The abscopal effect has a close relation with the discussed rationale of systemic immune response induced by IR above which includes the infiltrating immune cells and reprogrammed tumor microenvironment. Local IR triggers systemic antitumor immune response through these rationales, so next we will focus on the alteration of non-irradiated sites in molecule and gene level.

The molecular abscopal effect mainly refers to the alteration of cytokines and chemokines induced by IR at distant non-irradiated sites. There are two conditions: high-dose RT or low-dose RT. High-dose RT causes immunogenic cell death and leads to a systemic antitumor response. We have already certified the changes of cytokines and chemokines including TNFα, IL-1, IL-6, MCP-1, IL-8 at irradiated sites^34^ and that IR forms inflammatory tumor microenvironment remodeling by tumur cells.^95^ However, little is known about the expression levels of these immunomodulatory factors in tissues distant from the irradiated zone. Siva et al. proved that unirradiated out-of-field tissues exhibited delayed “abscopal” DNA damage response.^329^ This may induce a series of response to the DNA damage and change distant tumor microenvironment at abscopal sites. Similarly, Ventura et al.^330^ observed oxidatively induced clustered DNA lesions and apoptotic cell death were elevated in a wide variety of unirradiated tissues and these events were accompanied by changes in plasma concentrations of cytokines including macrophage-derived cytokine, eotaxin, IL10, VEGF, TGFβ1, and TGFβ2. These alterations not only exhibit RT toxicities as a cytotoxic agent but also shows potential to mediate abscopal effect by reshaping the tumor environment in many ways.^331,332^ One of the possible mechanisms of is the immunomodulatory effect by tumor-derived exosomes. Tumor-derived exosomes contain genetic materials and also carry immunomodulatory molecules. These tumor-derived exosomes may carry these signals to distant sites and exert an interaction with other immune cells, leading to an abscopal effect.^333^

Moreover, expression levels of cytokines and chemokines in plasma make a difference after treatment with definitive intensity-modulated RT.^334^ Abscopal gene expression in the out-of-field skin after synchrotron RT was monitored in which gene expression levels of TNF and TGFβ1 increased indicating a systemic inflammatory response and there was a decrease in Ccl2, Mdm2, and Trp53 gene expression.^335^ Similarly, Aravindan et al. found a robust increase in p65 and cMyc expression in distant heart after the radiation of lower abdomen.^336^ These results indicate that the status of abscopal sites has possibly started to change before the observed tumor regression. Hence, the alteration of non-irradiated sites in molecule and gene level may reflect the sensitivity of patients to RT and become predictors of abscopal effect and response to the treatment. For instance, recent studies indicated that tumor-derived exosomes may become a biomarker in gastrointestinal cancer.^337^ The contents of exosomes also had a strong association with survival.^338^ However, the clinical meanings of molecular and genetic changes of abscopal sites induced by RT remains unclear for the lack of clinical data. We expect more investigations to explore the molecular and genetic changes of abscopal sites to benefit more patients.

Fortunately, the authors observed the effect of low-dose RT on non-irradiated tumor stroma and discussed possible mechanisms and values.^280^ Combined with SBRT and immunotherapy, low-dose RT (doses below the threshold thought to physically damage DNA or kill cancer cells directly) stimulated abscopal tumor stroma throughout the body and facilitated abscopal tumor regression. This provides circumstantial evidence for the existence of molecular abscopal effect and thereout it triggers new thoughts that based on pre-existing abscopal effect in molecule level, low-dose radiation and immunotherapy offer a force more powerful method to activate abscopal antitumor immune response causing macroscopic tumor regression. Moreover, Yin et al.^321^ discussed about potential mechanisms of low-dose RT. In summary, both high-dose and low-dose radiation can mediate molecular alteration at abscopal tumor site and exert systemic antitumor response. However, mechanisms about how molecular changes of abscopal sites remains largely unclear and what happened at abscopal sites in the condition of high-dose radiation is unknown. We expect there is much direct and powerful evidence to explore the abscopal effect at the molecular level and we hold the view that this will increase occurrence rate of abscopal effect and provide new approach to overcome the resistance of immunotherapy.

### Immunomodulatory effect of low-dose radiation and radscopal effect

With a view to immune effect of conventional dose RT, low-dose RT may play a distinct role in antitumor immune response combined with IO. Many studies have affirmed the immunomodulatory effect of low-dose RT,^220,339–341^ sometimes termed the “radscopal effect”. Radscopal effect refers to the systemic antitumor effects which are strengthened by low-dose RT on the basis of stereotactic RT. This radiation strategy with high-dose stereotactic RT and low-dose RT was proposed by James Welsh and this strategy was named as “RadScopal” technique.^342^ Unlike tumoricidal-dose RT, low-dose RT can reprogram the tumor microenvironment and reactivate the immune microenvironment, thus reversing the resistance of patients to IO. Herrera et al. also reviewed that innate and adaptive immunity can be mobilized when all lesions are treated with low-dose RT combined with IO.^343^ Generally, conventional RT may produce potent immunosuppressive factors but low-dose radiation may be a strong weapon to address these limitations. We have discussed the immunomodulatory effects of IR in the above part. Of note, low-dose RT shows a similar but distinguishing effect on human immune system. The underlying mechanisms of distinguishing effect of low-dose RT may be initiated from DNA damage. Low-dose RT may lead to various lesions of DNA. DNA damage after such low doses is not sufficiently severe to induce cells death but can initiate danger signaling.^344^ As we discussed in the part of “Effects of IR on cancer cells”, defects of DNA repair may activate cGAS-STING pathway which induces inflammatory response. In addition, DAMPs released from these damaged cells may further activate immune responses.

However, there are few studies to investigate the effect of low-dose RT on immune cells. A large body of evidence has implicated that low-dose RT may result in the damage of CD4^+^ T cells and promote the conversion to a Th2 phenotype.^345^ Just like we discussed above, low-dose RT leads to generation of an iNOS^+^/M1 phenotype and these iNOS^+^/M1 macrophages can orchestrate effective T cell recruitment and kill tumor cells through iNOS.^220^ Yu and colleagues found that in vitro when DCs are exposed to 0.2 Gy radiation, there is an increase of migration mediated by CCR7 and IL-12 production induced by the ATM/NF-κB pathway.^269^ Persa et al.^346^ showed that low and high-dose irradiation-induced qualitatively different functional changes in murine splenic DCs in vivo. They found that low-dose RT stimulated antigen uptake and lowered antigen presentation while high doses did not influence. A recent study showed that targeted radionuclide therapy can render immunologically cold syngeneic B78 melanoma tumors sensitive to ICIs.^347^ Researchers observed a significant increase in tumor-infiltrating myeloid (CD11b^+^), and NK cells and an increase in the ratio of effector CD8^+^ to suppressor Treg cells after low-dose RT. Moreover, Herrera et al.^348^ found that low-dose RT reprogrammed the tumor microenvironment of tumors with scarce immune infiltration and elicited predominantly CD4^+^ cells with features of exhausted effector cytotoxic cells, with a subset expressing NKG2D and exhibiting proliferative capacity, as well as a unique subset of activated dendritic cells expressing the NKG2D ligand Rae1. Interestingly, low-dose splenic radiation may reduce CTLA-4 expression on the Treg cell surface to inhibit liver tumor development and researchers observed a significant decrease of the percentage of CD4^+^CD25^+^Treg/CD4^+^ cells in the blood and the expressions of Foxp3, IL-10, TGF-β, and CTLA-4 in spleen and liver tumors in this diethylnitrosamine-induced rat liver tumor model.^349^ Low-dose RT also sensitizes tumor cells to immune rejection by locally activated CAR T cells. In a model of pancreatic adenocarcinoma heterogeneously expressing sialyl Lewis-A (sLeA), DeSelm and colleagues found that not only sLeA+ but also sLeA− tumor cells exposed to low-dose RT become susceptible to CAR therapy, reducing antigen-negative tumor relapse.^350^

These results show potential prospect of low-dose radiation to promote antitumor response which could be called radscopal effect. In consideration of low occurrence of abscopal effect, there are several possible mechanisms: the insufficient immune components to exert effective antitumor response and the inhibitory immune barriers caused by tumors at abscopal site. Low-dose RT can overcome these limitations exactly. On the one hand, low-dose RT can increase secretion of chemokines involved in the attraction of T cells (e.g., CXCL9, CXCL10, CXCL11, CCL4, and CCL5).^321^ Thus, the CD8^+^ T cells primed by primary RT could be attracted to the remote secondary tumor and low-dose RT amplifies the potential of abscopal effect. Besides, low-dose RT can reprogram the tumor microenvironment by increasing T cells, NK cells, polarizing M1 macrophage, upregulating immunostimulatory factors such as NKG2D and its ligand and downregulating inhibitory factor.^342,348^ Figuratively speaking, abscopal effect of RT increases the number of soldiers (immune cells and effective molecules) but sometimes their force is insufficient to enter the city. However, the radscopal effect of low-dose RT gathers soldiers together and opens the city gates to bring the soldiers into the city.

Radscopal effect has been proven in preclinical and clinical studies. In mice with lung adenocarcinoma tumors, Barsoumian and his colleagues observed low-dose RT improved the outcomes of ICIs and identified potential mechanisms by which low-dose radiation promotes M1 macrophage polarization, enhances NK cells infiltration and reduces TGF-β levels.^342^ In a phase 2 prospective trial, it is reported that the combination of high and low-dose RT limits tumor growth at primary and secondary sites.^351^ Furthermore, in a phase II trial of SBRT and ipilimumab, researchers found that lesions receiving low-dose radiation were more likely to respond to the combined therapy.^352^ In addition, a recent clinical trial reported that LDRT plus high-dose RT safely improved lesion-specific response in patients with immune-resistant solid tumors by promoting infiltration of effector immune cells into the tumor microenvironment which is consistent with previous data.^353^ In a phase I clinical trial administering low-dose RT, low-dose cyclophosphamide and immune checkpoint blockade to patients with immune scarce tumors, researchers observed that the combinatorial treatment triggered T-cell infiltration, predominantly of CD4^+^ cells with Th1 signatures.^348^ Similarly, Yin et al.^321^ proved that hypo-fractionated RT of the primary tumor plus low-dose RT of the abscopal tumor as well as administering PD1 blockades enhances the abscopal response and the triple therapy group achieved optimal abscopal tumor growth control.

Thus, we believe that low-dose RT will further improve regimen of iRT and benefit more people from iRT as a trigger of systemic antitumor immune response.

## Biomarkers of treatment response

Although iRT has shown advantages in many kinds of tumors, just part of patients can get benefit from the combination. Thus, it is crucial and urgent to investigate efficient and precise biomarkers which predict and evaluate response to treatment. Besides, validated biomarkers may promote the selection of patients and prediction of outcomes. So far, some biomarkers for IO alone have been widely tested including tumor mutation burden, DNA repair deficiencies, PD-L1 expression, and gut microbiome.^354–356^ For instance, several studies reported that the higher tumor mutation burden is associated with better outcomes of ICIs.^357,358^ However, due to the later response of IO and several limitations, there is a lack of evidence to support them as predictors of iRT. It is necessary to find novel biomarkers or further explore the efficacy of former biomarkers.

In view of the rational of iRT, DAMPs reflecting the key events of RT-induced immunogenic cell death might be potential biomarkers to predict the efficacy of iRT. A large body of evidence has shown that RT leads to immunogenic cell death by upregulating calreticulin, as one of DAMPs.^359,360^ In addition, calreticulin induced by RT may play a key role in uptake tumor cells and augmenting immune cells.^48^ Thus, the level of calreticulin after RT may indicate the sensitivity of tumor cells to T cells and it has the potential to act as a biomarker of iRT.

Absolute lymphocyte count (ALC) may serve as another biomarker to predict abscopal effect. The circulating lymphocyte population plays as the most crucial component in the anticancer immune response, governing immune response to RT as well as response to ICIs. Early clinical data suggest that higher lymphocyte counts are associated with a higher response rate and more durable treatment response in patients treated with ICIs.^361^ In an analysis of 165 patients from three prospective trials evaluating the combination of RT and IO, investigators found that pre-RT ALC was significantly associated with progression-free survival (PFS) in both the traditional RT and SBRT groups.^362^ In a study with 11 patients administering ipilimumab after RT, Grimaldi et al. found that the occurrence of abscopal effect is associated with the median ALC before RT.^363^ This result also corroborates another study using data from 3 institutional phase 1/2 trials to examine the predictive capacity of recorded parameters in patients undergoing combined RT and IO. They found that post-RT absolute lymphocyte count, when analyzed as a continuous variable, correlated with abscopal responses.^364^ For post-RT ALC, the abscopal response rate was 34.2% in the cohort with ALC higher than the median value, compared with 3.9% in patients with ALC lower than the median. These data indicate the predictive role of ALC on systemic abscopal effects induced by iRT.

## Clinical practice

Based on the significant immunomodulatory effect of IR, we may broadly apply the use of iRT. In fact, iRT has been a great success to treat patients with cancers including NSCLC, melanoma, and some solid tumors.^365–371^ Compared with monotherapy, iRT shows significant perspectives to the local control of lesion sites and abscopal effect. Here we systematically summarize the clinical trials of iRT in recent years and explore the potential value in clinical application. Since the initial proof-of-principle trial about iRT, Golden and his colleagues first proved that RT combined with granulocyte-macrophage colony-stimulating factor (GM-CSF) does produce a high objective abscopal effect in patients who are suffering from metastatic solid tumors and this trial provides the proof-of-principle for the next clinical trials using iRT to treat metastatic tumors.^70^ Based on the evidence of KEYNOTE-001 for efficacy and safety of pembrolizumab, Shaverdian et al.^372,373^ further investigated that patients with advanced NSCLC treated with RT previously get longer PFS and OS with pembrolizumab than patients who did not receive previous RT, with an acceptable safety profile. And this study was the largest to report the effects of previous RT on the activity and toxicity of ICIs at the time. In addition to PD-1/PD-L1 inhibitors, the combination of RT with CTLA-4 inhibitors has also been shown to have an abscopal effect and therapeutic effect. In addition, Formenti et al.^374^ found that for patients with NSCLC who have not responded to IO before, some patients respond to IO, and some respond with significantly longer survival time when they receive treatment of RT combined with CTLA-4 blockade. The most powerful evidence supporting iRT was the phase III PACIFIC trial, which observed that the median PFS of patients with locally advanced, unresectable NSCLC is 16.8 months with the PD-L1 antibody durvalumab versus 5.6 months of patients with placebo.^42^ Furthermore, they found that durvalumab significantly prolonged overall survival, as compared with placebo and the following results including patient-reported outcomes, 3-year survival rates and 4-year survival rates confirmed that a clinical benefit can be attained through IO combined with chemoradiotherapy in PACIFIC study indeed.^43–45,375^ Based on Pembro-RT trial, a pooled analysis of two randomized trials from the Netherlands and MD Anderson including Pembro-RT showed that the effect of RT combined was statistically valid in advanced NSCLC.^376,377^ However, the choice of ICIs and RT including agents, sequence, dose, fractionation, and irradiated sites to exert the best synergies needs to be explored and optimized.^378^ Hereafter, we attempt to summarize the existing clinical trials to look at the possible optimal combination of iRT and the broad prospect of iRT to treat locally advanced and advanced tumors.

### Radiotherapy dose/fractionation

Concerning the rationale and some crucial clinical trials, the preponderance of iRT has been established. However, there are still many questions worth to be discussed such as the dose, lesion selection, and scope of RT. As we discussed above, the immune response induced by RT is “dose dependent” so the optimal dose should maximize tumor immunity and could be tolerant by patients. A preclinical study reported that RT in 7.5 Gy/fraction may achieve a better outcome along with maintaining low Treg numbers in mice bearing B16-ovalbumin murine melanoma, but not the 5 Gy.^379^ Another similar study showed the use of 15 Gy single-dose irradiation resulted in a greater number of host immune cells infiltrating tumors, compared with the 3 Gy × 5 fractionated schedule^261^. However, high-dose RT (≥15 Gy) may increase the proportion of splenic Tregs suppressing the antitumor immune response.^380^ In addition, other preclinical studies favored that conventional fractionation might have better efficacy to combine with IO.^111^ In clinical practice, it seems that hypofractionated RT may show advances in some certain cancers and the combination of IO + SBRT may be more potential in the modality of iRT.^71^ In the subgroup analysis of PACIFIC study, the investigator has proved that patients may get a dramatical survival benefit no matter what radiation doses are used.^381^ In a randomized phase I/II trial for lung and liver lesions of NSCLC,^382^ there were better out-of-field ORRs and longer median PFS times of pembrolizumab + SBRT (50 Gy in 4 fractions) compared with pembrolizumab + traditional RT (45 Gy in 15 fractions) which may indicate that hypofractionated radiotherapy can better coordinate the effect of immunotherapy. In the concurrent pembrolizumab + RT groups, the out-of-field ORRs were 38% in the pembrolizumab + SBRT group and 10% in the pembrolizumab + traditional RT group. This result also corroborates other clinical trials.^383^ Nevertheless, SBRT with PD-1 inhibitor and low-dose cyclophosphamide showed no significant clinical benefit compared with conventional RT in patients with metastatic colorectal cancer.^384^ The intriguing results of clinical trials may be associated with different tumor types and heterogeneity. In conclusion, these data show SBRT and, hypofractionated RT may exert a more effective antitumor immune response but the optimal dose for patients still needs more clinical data to identify. And there are a large body of clinical trials in this field (Table 2). The data from these ongoing clinical trials may contribute to the selection of RT modalities.

**Table 2:** Representative trials using combination of radiotherapy and immunotherapy

ClinicalTrials.gov identifier	Trial Phase	Condition or disease	Sequence	RT	IO	Results	Sponsors	Estimated/actual study completion date
NCT02474186	Phase 1 Phase 2	Various	Concurrent	35 Gy in 10 fractions	GM-CSF	Abscopal responses in 27.6% of patients	NYU Langone Health	July 2015
NCT02125461	Phase 3	NSCLC	RT, IO	54 to 66 Gy	Durvalumab	Durable PFS and sustained OS benefit with durvalumab after chemoradiotherapy	AstraZeneca	December 30, 2022
NCT02608385	Phase 1	Solid tumors	SBRT, IO	SBRT dosing varied by site and ranged from 30 to 50 Gy in three to five fractions	Pembrolizumab	Well tolerated with acceptable toxicity	University of Chicago	July 2022
NCT02221739	Phase 1 Phase 2	NSCLC	Concurrent	6 Gy x5, later changed to 9.5 Gy x3	Ipilimumab	Objective responses were observed in 18%, and 31% had disease control	NYU Langone Health	October 27, 2015
NCT02434081	Phase 2	NSCLC	Concurrent	66 Gy in 33 fractions	Nivolumab	The addition of nivolumab to concurrent CRT is safe and tolerable	European Thoracic Oncology Platform	March 31, 2020
NCT02492568	Phase 2	NSCLC	RT, IO	SBRT 3 doses of 8 Gy	Pembrolizumab	Well tolerated and a doubling of ORR	The Netherlands Cancer Institute	June 2018
NCT02444741	Phase 1 Phase 2	NSCLC	Concurrent	Various	Pembrolizumab	Safe and more beneficial for patients with low PD-L1 expression	M.D. Anderson Cancer Center	September 17, 2022
NCT02343952	Phase 2	Carcinoma, NSCLC	RT, IO	59.4 to 66.6 Gy	Pembrolizumab	PFS and OS improvement with consolidation pembrolizumab	Nasser Hanna, M.D.	September 2022
NCT03631784	Phase 2	NSCLC	Concurrent	60 Gy in 30 daily fractions	Pembrolizumab	Promising antitumor activity and manageable safety	Merck Sharp & Dohme Corp.	May 15, 2023
Ongoing or completed clinical trials using combination of radiotherapy and immunotherapy for NSCLC								
ClinicalTrials.gov identifier	Trial Phase	Condition or disease	Sequence	RT	IO	Status	Sponsors	Estimated/Actual study completion date
*PD-1 inhibitor*								
NCT03035890	Not Applicable	Metastatic NSCLC	Concurrent	Hypo-fractionated Radiation	Immuno-Therapeutic Agent (Nivolumab/ pembrolizumab/ atezolizumab)	Active, not recruiting	West Virginia University	June 30, 2023
NCT05111197	Phase 3	Locally advanced or metastatic NSCLC	IO, RT	SBRT	Anti-PD-1 or anti-PD-L1 immunotherapy	Not yet recruiting	Institut Cancerologie de l’Ouest	December 2024
NCT03523702	Phase 2	Locally Advanced NSCLC	Concurrent	Selective personalized radiotherapy	Pembrolizumab	Recruiting	Albert Einstein College of Medicine	September 2022
NCT03383302	Phase 1 Phase 2	NSCLC Stage II and Stage I	RT, IO	SBRT	Nivolumab	Recruiting	Royal Marsden NHS Foundation Trust	January 2022
NCT03825510	Not Applicable	Metastatic NSCLC	RT, IO	SBRT	Nivolumab/ pembrolizumab	Recruiting	Crozer-Keystone Health System	August 28, 2021
NCT04577638	Phase 2	NSCLC Stage III	Concurrent	Intensity Modulated Radiotherapy	Nivolumab	Recruiting	Center Eugene Marquis	February 1, 2024
NCT03168464	Phase 1 Phase 2	NSCLC Metastatic	Concurrent	6 Gy x 5 fractions	Ipilimumab/ nivolumab	Recruiting	Weill Medical College of Cornell University	December 30, 2022
NCT03867175	Phase 3	Stage IV NSCLC	Concurrent	SBRT	Pembrolizumab	Recruiting	Wake Forest University Health Sciences	December 31, 2027
NCT03110978	Phase 2	Stage I-IIA or Recurrent NSCLC	Concurrent	SBRT	Nivolumab	Recruiting	M.D. Anderson Cancer Center	June 30, 2022
NCT03812549	Phase 1	Stage IV NSCLC	RT, IO	SBRT/LDRT	Sintilimab	Recruiting	Sichuan University	December 31, 2022
NCT03313804	Phase 2	NSCLC, Squamous Cell Carcinoma of the Head and Neck	IO, RT	SBRT	Nivolumab/ pembrolizumab/ atezolizumab	Recruiting	John L. Villano, MD, PhD	June 30, 2028
NCT04929041	Phase 2 Phase 3	Stage IV NSCLC	Concurrent	SBRT	Ipilimumab/nivolumab/ pembrolizumab	Recruiting	National Cancer Institute (NCI)	December 31, 2027
NCT03774732	Phase 3	NSCLC Metastatic	Concurrent	SBRT	Pembrolizumab	Recruiting	UNICANCER	September 21, 2024
NCT05229614	Phase 2	NSCLC, Head and Neck Squamous Cell Carcinoma, Melanoma, Urothelial Carcinoma	IO, RT	Carbon ion therapy	Pembrolizumab	Not yet recruiting	CNAO National Center of Oncological Hadrontherapy	August 2026
NCT03705806		Stage IV NSCLC	IO, RT	30 Gy in 10 fractions	PD-1 inhibitor	Recruiting	University Health Network, Toronto	September 15, 2022
NCT03224871	Early Phase 1	Metastatic NSCLC	Concurrent	Hypo-fractionated Radiotherapy	Intralesional IL-2, nivolumab, pembrolizumab	Completed	University of California, Davis	January 10, 2020
NCT05265650	Phase 1 Phase 2	Metastatic NSCLC	Concurrent	SBRT	Nivolumab	Not yet recruiting	Clinica Universidad de Navarra, Universidad de Navarra	June 2024
NCT04513301	Phase 2	Recurrent or IV NSCLC after failure of platinum-based chemotherapy	Concurrent	50-60 Gy/25-30 f	Sintilimab	Recruiting	Shanghai Cancer Hospital, China	December 1, 2022
NCT05222087	Phase 1	Metastatic NSCLC	RT, IO	SBRT	Pembrolizumab	Not yet recruiting	Peter MacCallum Cancer Centre, Australia	April 2024
NCT02444741	Phase 1 Phase 2	Stage IV NSCLC	Concurrent	SBRT	Pembrolizumab	Active, not recruiting	M.D. Anderson Cancer Center	September 17, 2022
NCT03217071	Phase 2	Stage I-IIIA NSCLC	IO, RT	SBRT	Pembrolizumab	Active, not recruiting	Sue Yom	February 28, 2022
NCT02492568	Phase 2	Advanced NSCLC	RT, IO	SBRT 3 doses of 8 Gy	Pembrolizumab	Completed	The Netherlands Cancer Institute	June 2018
NCT04892849	Not Applicable	HNSCC, NSCLC, Esophageal Cancer, Urothelial Carcinoma, Renal Cell Carcinoma, Squamous Cell Carcinoma of the Skin, Small Cell Bronchial Carcinomas	IO, RT	RT	PD-1/PD-L1 inhibitor	Recruiting	University of Erlangen-Nürnberg Medical School	December 31, 2027
NCT03223155	Phase 1	Stage IV NSCLC	Concurrent or sequential	SBRT	Ipilimumab/ nivolumab	Recruiting	University of Chicago	December 2024
NCT04013542	Phase 1	Stage II–III NSCLC	Concurrent	RT	Ipilimumab and nivolumab	Recruiting	M.D. Anderson Cancer Center	February 1, 2022
NCT04902040	Phase 1 Phase 2	Advanced Bladder Carcinoma, Advanced NSCLC, Advanced Malignant Solid Neoplasm, Advanced Melanoma, Advanced Merkel Cell Carcinoma, Advanced Renal Cell Carcinoma	RT, IO	RT	Atezolizumab, avelumab, durvalumab, nivolumab and pembrolizumab	Recruiting	M.D. Anderson Cancer Center	June 1, 2025
NCT04271384	Phase 2	Stage 1 NSCLC	Concurrent	SBRT	Nivolumab	Recruiting	Hospital Israelita Albert Einstein	June 29, 2023
NCT04291092	Phase 2	NSCLC Stage IV Brain Metastases		WBRT	Camrelizumab	Recruiting	Zhejiang Cancer Hospital	June 30, 2023
NCT04167657	Phase 2	Advanced NSCLC	RT, IO	RT	Sintilimab	Recruiting	Peking Union Medical College Hospital	April 15, 2023
NCT02818920	Phase 2	Stage IB, II or IIIA NSCLC	IO, RT	RT	Pembrolizumab	Active, not recruiting	Neal Ready	March 2026
NCT03589339	Phase 1	Advanced cancers (Metastatic NSCLC)	IO, RT	SBRT	Nivolumab/ pembrolizumab	Recruiting	Nanobiotix	March 30, 2023
NCT04977453	Phase 1 Phase 2	NSCLC, Head and Neck Squamous Cell Carcinoma, Renal Cell Carcinoma, Urinary Bladder Cancer, Melanoma, Sarcoma		SBRT	Pembrolizumab	Recruiting	GI Innovation, Inc.	December 2025
*PD-L1 inhibitor*								
NCT03965468	Phase 2	NSCLC Stage IV Oligometastasis	Concurrent	SBRT	Durvalumab	Recruiting	European Thoracic Oncology Platform	December 2021
NCT03035890	Not Applicable	Metastatic NSCLC	Concurrent	Hypo-fractionated Radiation	Immuno-Therapeutic Agent (Nivolumab/ pembrolizumab/ atezolizumab)	Active, not recruiting	West Virginia University	June 30, 2023
NCT05000710	Phase 2	Metastatic or Locally Advanced NSCLC	Concurrent	11 fractions of 3 Gy	Durvalumab/ tremelimumab	Recruiting	Sheba Medical Center	December 2026
NCT05111197	Phase 3	Locally advanced or metastatic NSCLC	IO, RT	SBRT	Anti-PD-1 or anti-PD-L1 immunotherapy	Not yet recruiting	Institut Cancerologie de l’Ouest	December 2024
NCT04765709	Phase 2	Large volume stage III NSCLC	Concurrent	RT	Durvalumab	Not yet recruiting	Mario Negri Institute for Pharmacological Research	June 2026
NCT04549428	Phase 2	NSCLC Stage IV	Concurrent	a single fraction of 8 Gy	Atezolizumab	Recruiting	Oncology Institute of Southern Switzerland	July 31, 2022
NCT04245514	Phase 2	NSCLC	Concurrent	20 × 2 Gy (weekdaily, 4 weeks) 5 × 5 Gy (weekdaily, 1 week) 3 × 8 Gy (on alternate days, 1 week)	Durvalumab	Recruiting	Swiss Group for Clinical Cancer Research	March 2025
NCT05267392	Phase 1 Phase 2	Early stage or locally advanced, unresectable NSCLC	IO, RT	Standard of care RT/RCT	Durvalumab	Recruiting	Instituto Portugues de Oncologia, Francisco Gentil, Porto	January 2024
NCT05128630	Phase 2	NSCLC, Stage III	Concurrent	reduced-dose hypo-fractionated thoracic RT	Durvalumab	Recruiting	IRCCS Policlinico S. Matteo	November 28, 2025
NCT04989283	Phase 2	Stage IIB Lung Cancer AJCC v8, Stage IIIA Lung Cancer AJCC v8, Superior Sulcus Lung Carcinoma	Concurrent	External Beam Radiation Therapy	Atezolizumab	Recruiting	National Cancer Institute (NCI)	May 10, 2031
NCT03313804	Phase 2	NSCLC, Squamous Cell Carcinoma of the Head and Neck	IO, RT	SBRT	Nivolumab/ pembrolizumab/ atezolizumab	Recruiting	John L. Villano, MD, PhD	June 30, 2028
NCT03275597	Phase 1	NSCLC Stage IV	IO, RT	SBRT	Durvalumab + tremelimumab	Active, not recruiting	University of Wisconsin, Madison	July 2025
NCT04372927	Phase 2	Locally Advanced NSCLC	Concurrent	Adaptive mediastinal radiation	Durvalumab	Recruiting	University of Washington	November 30, 2026
NCT03916419	Phase 2	Stage IIB, IIIA, and Select IIIB and IIIC NSCLC	Concurrent	MR-Linear Accelerator-Radiation	Durvalumab	Recruiting	Washington University School of Medicine	December 31, 2024
NCT04230408	Phase 2	Stage III NSCLC	IO, RT, IO	54 to 66 Gy	Durvalumab	Recruiting	Latin American Cooperative Oncology Group	May 2024
NCT04992780	Phase 2	NSCLC	RT, IO	Hypo-Fractionation 62.5 Gy in 25 fractions of 2.5 Gy/fraction; Standard-Fractionation 60 Gy in 30 fractions of 2 Gy/fraction	Durvalumab	Not yet recruiting	University of Kansas Medical Center	November 2023
NCT03446547	Phase 2	Stage I NSCLC	RT, IO	SBRT	Durvalumab	Recruiting	Vastra Gotaland Region	July 2023
NCT05034055	Phase 2	Metastatic NSCLC	RT, IO	SBRT	Atezolizumab/tiragolumab	Not yet recruiting	Yonsei University	December 2023
NCT05157542	Phase 1	Stage III NSCLC	Concurrent	Low dose radiation therapy	Durvalumab	Recruiting	Juan LI, MD	June 10, 2023
NCT03391869	Phase 3	Stage IV NSCLC	IO, RT	Local consolidation therapy	Nivolumab and ipilimumab	Recruiting	M.D. Anderson Cancer Center	December 31, 2022
NCT03818776	Early Phase 1	Unresectable NSCLC	Concurrent	Proton beam therapy RT	Durvalumab	Recruiting	Case Comprehensive Cancer Center	November 1, 2023
NCT03801902	Phase 1	Locally advanced NSCLC	IO, RT	Hypofractionated Radiation Therapy/Fractionated Stereotactic Radiation Therapy	Durvalumab	Active, not recruiting	National Cancer Institute (NCI)	January 5, 2023
NCT02463994	Early Phase 1	Metastatic NSCLC	RT, IO	Hypo-fractionated Image-guided Radiotherapy	PD-L1 antibody	Completed	University of Michigan Rogel Cancer Center	November 7, 2018
NCT02888743	Phase 2	Metastatic Colorectal or NSCLC	IO, RT	high dose radiation therapy/low dose radiation therapy	Tremelimumab and durvalumab	Active, not recruiting	National Cancer Institute (NCI)	December 31, 2022
NCT04944173	Phase 2	Stage I NSCLC	Concurrent	SBRT	Durvalumab	Not yet recruiting	University of British Columbia	December 2024
NCT04310020	Phase 2	Stage II or III NSCLC	IO, RT	Hypo-fractionated Radiation Therapy	Atezolizumab	Recruiting	National Cancer Institute (NCI)	March 15, 2022
NCT04081688	Phase 1	Refractory NSCLC Stage IV		SBRT	Atezolizumab/ varlilumab	Recruiting	Rutgers, The State University of New Jersey	June 30, 2023
NCT04889066	Phase 2	Brain metastases NSCLC	Concurrent	Personalized ultra-fractionated stereotactic adaptive radiotherapy or Fractionated Stereotactic Radiotherapy	Durvalumab	Not yet recruiting	University of Texas Southwestern Medical Center	January 2025
NCT04892849	Not Applicable	HNSCC, NSCLC, Esophageal Cancer, Urothelial Carcinoma, Renal Cell Carcinoma, Squamous Cell Carcinoma of the Skin, Small Cell Bronchial Carcinomas	IO, RT	RT	PD-1/PD-L1 inhibitor	Recruiting	University of Erlangen-Nürnberg Medical School	December 31, 2027
NCT04202809	Phase 2	Resectable Stage III NSCLC	Concurrent	RT	Durvalumab	Recruiting	University Hospital, Essen	April 2024
NCT03237377	Phase 2	Stage III Resectable NSCLC	Concurrent	Thoracic radiation: 45 Gy in 25 fractions	Durvalumab or durvalumab plus tremelimumab	Active, not recruiting	Sidney Kimmel Comprehensive Cancer Center at Johns Hopkins	September 2022
NCT04214262	Phase 3	Stage I-IIA NSCLC	Induction/Consolidation Atezoli-zumab + SBRT	SBRT	Atezolizumab	Recruiting	National Cancer Institute (NCI)	May 1, 2028
NCT03871153	Phase 2	Stage III NSCLC	Concurrent	45-61.2 Gy/25-30 f	Durvalumab	Active, not recruiting	Greg Durm, MD	April 2023
NCT03141359	Phase 2	Locally advanced NSCLC	IO, RT	IMRT/SBRT	Durvalumab	Recruiting	Atrium Health	May 2026
NCT04902040	Phase 1 Phase 2	Advanced Bladder Carcinoma, Advanced NSCLC, Advanced Malignant Solid Neoplasm, Advanced Melanoma, Advanced Merkel Cell Carcinoma, Advanced Renal Cell Carcinoma	RT, IO	RT	Atezolizumab, avelumab, durvalumab, nivolumab and pembrolizumab	Recruiting	M.D. Anderson Cancer Center	June 1, 2025
NCT05198830	Phase 2	Stage III Non-Squamous NSCLC	RT, IO	RT	Durvalumab	Not yet recruiting	National Cancer Institute (NCI)	May 1, 2024
NCT04786093	Phase 2	Advanced NSCLC	Concurrent	SBRT/Personalized Ultra-fractionated Stereotactic Radiotherapy	Durvalumab	Recruiting	University of Texas Southwestern Medical Center	May 2027
NCT04364776	Not Applicable	Stage III Unresectable NSCLC	IO, RT	RT	Durvalumab	Recruiting	IRCCS Policlinico S. Matteo	April 15, 2024
NCT04892953	Phase 2	Stage III NSCLC	RT, IO	RT	Durvalumab	Not yet recruiting	M.D. Anderson Cancer Center	September 30, 2022
NCT02492867	Not Applicable	Locally Advanced NSCLC	RT, IO	Response-driven Adaptive Radiation Therapy	Durvalumab	Active, not recruiting	University of Michigan Rogel Cancer Center	November 2024
NCT04238169	Phase 2	Stage IV NSCLC	Concurrent	SBRT	Toripalimab	Recruiting	Xinqiao Hospital of Chongqing	December 31, 2023
NCT04597671	Phase 3	Stage III NSCLC	Concurrent	Low-dose prophylactic cranial irradiation	Durvalumab	Recruiting	Association NVALT Studies	December 2032
NCT03337698	Phase 1 Phase 2	Metastatic NSCLC	Concurrent	RT	Atezolizumab	Recruiting	Hoffmann-La Roche	August 1, 2025
NCT04092283	Phase 3	Unresectable Stage III NSCLC	IO, RT, IO	RT	Durvalumab	Recruiting	National Cancer Institute (NCI)	October 31, 2028
NCT05259319	Phase 1	Metastatic NSCLC, Metastatic Bladder Cancer, Metastatic Renal Cell Carcinoma, Metastatic Head and Neck Cancer	Concurrent or sequential	SBRT	Atezolizumab and tiragolumab	Not yet recruiting	Centre Georges Francois Leclerc	February 28, 2030
NCT03915678	Phase 2	Advanced solid tumors	Concurrent	SBRT	Atezolizumab	Recruiting	Institut Bergonié	March 2025
NCT03509012	Phase 1	Squamous Cell of Head and Neck Carcinoma, NSCLC	Concurrent	RT	Durvalumab/ tremelimumab	Active, not recruiting	AstraZeneca	December 29, 2023
*CTLA-4*								
NCT03168464	Phase 1 Phase 2	NSCLC Metastatic	Concurrent	6 Gy x 5 fractions	Ipilimumab/ Nivolumab	Recruiting	Weill Medical College of Cornell University	December 30, 2022
NCT04929041	Phase 2 Phase 3	Stage IV NSCLC	Concurrent	SBRT	Ipilimumab/ Nivolumab/ Pembrolizumab	Recruiting	National Cancer Institute (NCI)	December 31, 2027
NCT03275597	Phase 1	NSCLC Stage IV	IO, RT	SBRT	Durvalumab and tremelimumab	Active, not recruiting	University of Wisconsin, Madison	July 2025
NCT03391869	Phase 3	Stage IV NSCLC	IO, RT	Local consolidation therapy	Nivolumab and ipilimumab	Recruiting	M.D. Anderson Cancer Center	December 31, 2022
NCT02888743	Phase 2	Metastatic Colorectal or NSCLC	IO, RT	high dose radiation therapy/low dose radiation therapy	Tremelimumab and durvalumab	Active, not recruiting	National Cancer Institute (NCI)	December 31, 2022
NCT03223155	Phase 1	Stage IV NSCLC	Concurrent or sequential	SBRT	Ipilimumab/Nivolumab	Recruiting	University of Chicago	December 2024
NCT03237377	Phase 2	Stage III Resectable NSCLC	Concurrent	Thoracic radiation: 45 Gy in 25 fractions	Durvalumab or durvalumab plus tremelimumab	Active, not recruiting	Sidney Kimmel Comprehensive Cancer Center at Johns Hopkins	September 2022
NCT04013542	Phase 1	Stage II–III NSCLC	Concurrent	RT	Ipilimumab and nivolumab	Recruiting	M.D. Anderson Cancer Center	February 1, 2022
NCT02221739	Phase 1 Phase 2	Metastatic NSCLC	Concurrent	IMRT or 3-D CRT	Ipilimumab	Completed	NYU Langone Health	October 27, 2015
NCT03509012	Phase 1	Squamous Cell of Head and Neck Carcinoma, NSCLC	Concurrent	RT	Durvalumab/ tremelimumab	Active, not recruiting	AstraZeneca	December 29, 2023
*TIGIT inhibitor*								
NCT05034055	Phase 2	Metastatic NSCLC	RT, IO	SBRT	Atezolizumab/tiragolumab	Not yet recruiting	Yonsei University	December 2023
NCT05259319	Phase 1	Metastatic NSCLC, Metastatic Bladder Cancer, Metastatic Renal Cell Carcinoma, Metastatic Head and Neck Cancer	Concurrent or sequential	SBRT	Atezolizumab and tiragolumab	Not yet recruiting	Centre Georges Francois Leclerc	February 28, 2030
*Cytikines*								
NCT03705403	Phase 2	NSCLC Stage IV Metastatic Disease	Concurrent	SBRT	L19-IL2	Recruiting	Maastricht University	December 1, 2023
NCT03224871	Early Phase 1	Metastatic NSCLC	Concurrent	Hypo-fractionated Radiotherapy	Intralesional IL-2, nivolumab, pembrolizumab	Completed	University of California, Davis	January 10, 2020
Vaccines								
NCT00006470	Phase 2	NSCLC	IO, RT	RT	Monoclonal antibody 11D10 anti-idiotype vaccine or monoclonal antibody 3H1 anti-idiotype vaccine	Completed	Radiation Therapy Oncology Group	December 2004
NCT00828009	Phase 2	Unresectable Stage IIIA and IIIB Non-Squamous NSCLC	RT, IO	RT	Tecemotide	Completed	ECOG-ACRIN Cancer Research Group	May 22, 2019
*Others*								
NCT05269485	Phase 1 Phase 2	Stage III NSCLC		High-dose fractionated radiotherapy: 60–68 Gy/15-17f; low-dose fractionated radiotherapy: 48 Gy/15-12f	IT	Recruiting	Anhui Provincial Hospital	June 1, 2023
NCT04654520	Not Applicable	Stage IV NSCLC	Concurrent	IMRT	IT	Not yet recruiting	Guizhou Medical University	May 31, 2022
NCT04650490	Phase 2	Brain Metastases NSCLC	IO, RT or RT, IO	SBRT	IT	Not yet recruiting	Duke University	March 2025
NCT03827577	Phase 3	Oligometastatic NSCLC	RT, IO	SBRT	IT	Recruiting	Azienda Ospedaliera Universitaria Integrata Verona	September 2022
NCT02839265	Phase 2	Advanced NSCLC	Concurrent	SBRT	FLT3 Ligand Therapy (CDX-301)	Active, not recruiting	Albert Einstein College of Medicine	October 5, 2022
NCT04491084	Phase 1 Phase 2	Advanced NSCLC	Concurrent	SBRT	FLT3 ligand (CDX-301), anti-CD40 antibody (CDX-1140)	Recruiting	Albert Einstein College of Medicine	August 31, 2023
NCT00879866	Phase 1	NSCLC Stage IIIb With Malignant Pleural Effusion or Stage IV With Disease Control	RT, IO	5 ×4 Gy	Selectikine (EMD 521873)	Completed	Merck KGaA, Darmstadt, Germany	September 2012
NCT04081688	Phase 1	Refractory NSCLC Stage IV		SBRT	Atezolizumab/ varlilumab	Recruiting	Rutgers, The State University of New Jersey	June 30, 2023

The next question is lesion selection. Poleszczuk described a mathematical model that incorporates physiologic information about T-cell trafficking to estimate the distribution of focal therapy-activated T cells between metastatic lesions.^385^ Their study showed that not all metastatic sites participate in systemic immune surveillance equally and therefore the success in triggering the abscopal effect depends on the selection of metastatic site to receive the treatment. Likewise, an open-label, phase I trial determining SBRT and ipilimumab in patients with metastatic solid tumor refractory to standard therapies and ≥1 lesion in the liver or lung amenable to SBRT with ≥1 additional non-contiguous lesion for monitoring found that liver (vs. lung) irradiation produced greater T-cell activation, reflected as increases in the proportions of peripheral T cells expressing ICOS, GITR, and 4-1BB which is associated with better clinical benefit.^386^ Therefore, we need to consider how to select an appropriate lesion to activate immune response, or another way to think about it, we can use multi-site RT to achieve systemic disease control.^387–389^ The multi-site RT is based on our cognition to the most tumors and rationales of iRT. Achievement of abscopal effect depends on shared TAAs from an irradiated tumor recognized by other lesions, but it is precise because of the heterogeneity of tumors that not the entire cellular population can exert an effective immune response at these other lesions.^390,391^ Even for TAAs that could be recognized by entire cellular population, the immunosuppressive tumor microenvironment may be the barrier for CD8^+^ T cells to access the lesions.^392^ If we irradiate multiple lesions, or even all tumor lesions, it is possible to overcome these barriers to immune activation. As noted above, we can use low-dose radiotherapy to overcome immunosuppressive tumor microenvironment for access of CD8^+^ T cells and multi-site radiation might active more shared TAAs with the heterogeneity of different tumor lesions. Multiple retrospective studies in oligometastatic NSCLC have shown that the use of RT to all sites of disease is associated with a significant improvement in OS and PFS.^393,394^ Data from a phase III trial aiming to determine the efficacy of iRT in metastatic prostate cancer showed that results are negative when RT was delivered to a single bony lesion.^395^ Subgroup analyses of this trial and other clinical trials also support IO may achieve better efficacy when patients with lower disease burden and a reduction in tumor burden by comprehensive (but not single-site) RT may potentiate IO. Furthermore, a phase 2 trial of pembrolizumab therapy after locally ablative therapy (surgery or SBRT) for patients with oligometastatic NSCLC which treated all metastatic sites demonstrated that FPS increased by 12 months compared with the historical median of 6.6 months.^396^ Thus, multiple target RT might be required to optimize responses to iRT. Of note, a phase I study estimated multi-site SBRT followed by pembrolizumab for metastatic solid tumors, including NSCLC.^367^ This trial enrolled 79 patients receiving SBRT to 2 to 4 metastases and metastases >65 mL were partially irradiated. After completion of SBRT, patients started to administer pembrolizumab within 7 days and it lasted at least one cycle. The RECIST-based overall ORR was 13.2% and the mOS and mPFS were 9.6 months (95% CI, 6.5 months to undetermined) and 3.1 months (95% CI, 2.9 to 3.4 months), respectively. Their results are similar to data from KEYNOTE-028 (9–33% response rate), which evaluated the safety and efficacy of pembrolizumab alone in patients with PD-L1-positive advanced solid tumors.^397^ Nevertheless, investigators should consider these data carefully because the tumor areas excluded from the SBRT program are exposed to low doses of RT and there is a lack of PD-L1 status for patients with various cancers.

### Selection of immunotherapy modality

Although hypofractionated RT has shown advances for inducing antitumor immunity, it is not known which immune checkpoint inhibitor to use in combination with RT. There are an increasing number of studies which have already confirmed the efficacy of RT combined with ICIs including pembrolizumab, nivolumab, durvalumab, and atezolizumab.^42,398–401^ Considering that the most widely used ICI is PD-1 and CTLA-4 blockades, a retrospective analysis of two single-institution prospective trials reported that the FPS of anti-PD1 combined with SBRT for metastatic NSCLC was significantly better than anti-CTLA4 combined with SBRT, although there was no statistically significant difference in efficacy.^365^ The PFS was 76% for anti-CTLA4 vs 94% anti-PD1 at 3 months, 52% vs 87% at 6 months, 31% vs 80% at 12 months, and 23% vs 63% at 18 months (*p* = 0.02). This tentative exploration requires further data, and this conclusion is restricted to metastatic NSCLC. There are few of studies to support and more clinical trials are needed for other form of tumors. Notably, in the view of multi-site RT, we should consider whether we can use one or multiple ICIs. A randomized, multicenter, phase II clinical study assessing the primary safety of two kinds of ICIs as consolidation therapy after concurrent chemoradiotherapy for patients with stage III, unresectable NSCLC. This trial enrolled 105 patients with unresectable stage IIIA/ IIIB NSCLC and they were divided equally into to group (nivolumab group and nivolumab + ipilimumab group) after concurrent chemoradiotherapy. Safety analysis of the first 50 patients showed that after concurrent chemoradiotherapy, the incidence of grade 3 adverse events is higher in nivolumab + ipilimumab treatment, leading to higher drug withdrawal rate than that in nivolumab monotherapy group. Therefore, this area requires further exploration from additional clinical trials.

### The optimal timing for iRT

In addition to the selection of RT and immunotherapy molality, one of the most important matters is the optimal timing for combination “concurrent” or “sequential”.^402–405^ Generally, the data available to date seem to justify either simultaneous or delayed administration of checkpoint inhibitors after RT so that newly recruited T cells can destroy tumor cells, both at the primary site and systemically after being presented with novel tumor antigens. The PACIFIC trial has proved that durvalumab after chemoradiotherapy improved PFS significantly and the new results reported that estimated 4-year OS rates were 49.6% versus 36.3% for durvalumab versus placebo, and 4-year PFS rates were 35.3% versus 19.5% respectively.^42,45^ The PACIFIC trial has laid a framework of adjuvant administration of durvalumab after chemoradiotherapy for patients who are suffering from NSCLC in stage III. Similarly, other clinical trials also confirmed the safety and efficacy of pembrolizumab and nivolumab after chemoradiotherapy.^398,406^ However, the HOPE-005/CRIMSON, a multicenter, retrospective, real-world cohort study of 275 patients receiving concurrent chemoradiotherapy in advanced NSCLC, 204 of whom received durvalumab consolidation therapy showed that 81.8% of patients who received durvalumab after concurrent chemoradiotherapy had pneumonitis and 59.5% were asymptomatic pneumonitis.^407^ Another real-world study of durvalumab consolidation after chemoradiotherapy in stage III NSCLC suggested that the incidence of grade 3 radiation pneumonitis was 14.3 % in the durvalumab group versus 2.5 % in the observation group.^408^ These data remind that the adverse effect cannot be ignored and it needs to be further improved for PACIFIC mode. Analysis of PACIFIC study and a retrospective analysis of clinical data suggest that patient outcome seems to be better if IO is given concurrently or begun soon after RT, as compared with starting IO later after the RT.^42,409^ The update data from PACIFIC study show that there is a significant improvement of OS and FPS both within 14 days and after RT, but the advantage is more obvious within 14 days when combined IO with RT.^45^ However, a retrospective study proved that patients who received IO ≥ 21 days after the onset of SBRT had a longer OS than those who received IO within 21 days after the onset of SBRT.^410^ But the data from this study should be considered carefully because it included many confounding factors. Thus, the optimal timing of iRT still needs to be explored through large randomized clinical trials.

Sequential administration of IO followed by RT have been affirmed, many studies started to investigate whether we could use concurrent administration to achieve immunotherapy forward. Researchers hypothesized that adding IO concurrently with concurrent chemoradiotherapy may improve the efficacy without additive toxicity so they designed a phase II trial of concurrent administration.^411^ This phase II study was conducted in two parts. Part 1 involved administration of conventionally fractionated chemoradiotherapy followed by consolidation chemotherapy (atezolizumab [two cycles] and maintenance atezolizumab up to 1 year). Part 2 involved administration of concurrent chemoradiotherapy with atezolizumab followed by the same consolidation and maintenance therapies as in part 1. The results showed that immune-related adverse events of grade 3 or higher had an incidence rate of 20% to 30%, and pneumonitis of grade 2 or higher had an incidence rate of 10% to 16%. The median FPS of part 1/2 was 18.6 months and 13.2 months, respectively. Therefore, safety and efficacy of combining IO with chemoradiotherapy concurrently have been confirmed. Since the PACIFIC trial indicated that there was a trend of PFS being longer in the 14 days group, investigators designed a phase 2, nonrandomized KEYNOTE-799 clinical trial aiming for improve outcomes and safety of pembrolizumab with concurrent chemoradiotherapy.^46^ Patients corresponding to the inclusion criteria were selected by investigators to enter the following cohort: in cohort A, patients with squamous cell cancer/non-squamous cell cancer received paclitaxel 200 mg/m^2^ combined with carboplatin (AUC = 6), and after one cycle were switched to paclitaxel 45 mg/m^2^ combined with carboplatin (AUC = 2), lasting for 6 weeks. Two cycles of pembrolizumab therapy (once every three weeks) and standard radiotherapy (total dose was 60 Gy) were synchronized. In cohort B, patients with non-squamous cell cancer received pemetrexed (500 mg/m^2^) combined with cisplatin (75 mg/m^2^) and pembrolizumab (200 mg), combined with radiotherapy (60 Gy) in cycle 2 and 3. The results showed that ORR was 70.5% (79 of 112; 95% CI, 61.2–78.8%) in cohort A and 70.6% (72 of 102; 95% CI, 60.7–79.2%) in cohort B. Another coprimary end points, incidence of grade 3 to 5 pneumonitis, was 8.0% in cohort A and 6.9% in cohort B which were both less than 8.0% and meet expectation.^46^ A phase 1 trial compared combined nivolumab and ipilimumab with sequential or concurrent multi-site SBRT directly in patients with stage IV NSCLC.^412^ Their results showed that the median FPS of concurrent/sequential was 18.6 months and 13.2 months, respectively. Moreover, the concurrent group was no more toxic than sequential and there were no dose-limiting toxicity results in concurrent group but two patients had dose-limiting toxicity results in the sequential group. Thus, the data from this study proved that multi-site SBRT combined with nivolumab and ipilimumab concurrently had good safety and considerable efficacy. Similarly, a multicentric retrospective study from AIRO (Italian Association of Radiotherapy and Clinical Oncology) for patients with brain metastases from NSCLC reported that patients with the interval between SBRT and IO ≤ 7 days had a longer survival compared with the interval between SBRT and IO > 7 days.^413^ This study data supports the delivery of SBRT and IO within a short time frame given that concurrent therapy is shown to be more effective without having an impact on toxicity. In conclusion, these data from clinical trials and retrospective studies indicate that concurrent iRT may be more effective than sequential one. Nevertheless, due to relatively less clinical data, more studies are needed to investigate the optimal timing.

New opinions have been provided that neoadjuvant RT increases response rates and provides local control during neoadjuvant systemic treatment, before definitive surgery.^414^ Therefore, iRT may improve treatment responses and complete resection rates in the neoadjuvant setting for the better locally control and systemic antitumor responses. Besides, there are numerous ongoing clinical trials to optimize PACIFIC mode and strategies for IO forward such as KEYLYNK-012, CheckMate 73L, and PACIFIC-2 trials.^415–417^ We expect the data from these trials and believe in that the optimal combination will be established in the future.

In addition, other drugs also show enormous potential in cancer treatment and may promote the efficacy of iRT. Nanoparticles, as immunomodulators and radiosensitizers to overcome therapy resistance and improve survival, play a crucial role in cancer treatment.^418^ For the special properties, nanoparticles are an ideal carrier to enhance antitumor IO. It is reported that combining IO with nanoparticles may promote the accumulation and retention of antibodies in the target cells.^419^ For instance, nanoparticles-based antigens and adjuvants delivery strategies can address the issues of off-target side effect and low immunogenicity.^420^ Moreover, nanomaterials with heavy-metal showed a promising radiosensitization which can efficiently absorb, scatter, and emit radiation energy and redistribution cell cycle.^421,422^ Nanoparticles also can serve as delivery vehicles carrying the radiosensitivity drugs which shows a promising prospect.^423^ Of note, Wang et al. reported that cisplatin nanoparticles can promote the abscopal effect induced by RT with PD1 inhibitors which breaks through the efficiency limitation of iRT.^424^

Collectively, the authors have discussed the optimal combination modality for patients with metastatic NSCLC which may be hypofractionationated radiotherapy combined with anti-PD1 at the present and plenty of clinical trials have proved that the efficacy of iRT is significantly better than mono immunotherapy. The addition of radiotherapy increased the out-of-field response rate of immunotherapy alone from 19.7% to 41.7%.^376^ However, the optimal combinations that improve clinical outcomes for patients with tumors leave much for further study and we expect the results of ongoing clinical trials.

### The toxicity of iRT

Although iRT has made a breakthrough in both preclinical studies and clinical trials, it also raises the question of the toxicity of this combination strategy. It is well-documented that normal tissue injuries are the main limiting factor of the dose for RT alone. In view of the irradiated sites, these radiation-induced injuries include may include brain injury, heart disease, lung, and liver injury as well as damage to the corresponding sites.^425–431^ The most severe injury caused by RT is radiation-induced lung injury (RILI). RILI manifests as lung tissue damage and comprises two injury types: radiation pneumonitis and radiation pulmonary fibrosis.^428^ Generally, radiation pneumonitis occurs in 6 months after RT and radiation pulmonary fibrosis occurs >1 year following RT. The underlying mechanisms of RILI may include many signal pathways such as TGF-β/Smad, HMGB1/TLR4, and Nrf2/ARE signaling pathway as well as dysregulation of cytokines which are initiated from the DNA damage and ROS generation caused by RT. Another serious injury is radiation-induced heart disease (RIHD). It includes cardiomyopathy, conduction system abnormalities, coronary artery disease and the like. Similar to RILI, the pathogenesis of RIHD is associated with the production of cytokines induced by endothelial injury and oxidative stress.^426^ In addition, liver injury caused by RT cannot be neglected. Radiation-induced liver injury, which is different from RILI and RIHD, usually occurs during RT for some upper abdominal malignant tumors. In the clinic, radiation-induced liver injury can be classified into classic and non-classic liver disease.^432^ DNA damage and reactive free radical generation are two major factors to trigger liver injury caused by RT. There are various processes which are involved in radiation-induced liver injury and they finally cause liver cell apoptosis or necrosis.^430^ In addition to the direct damage of RT, mechanisms of RT‐induced normal tissue injury also involve a large number of immune cells and immunological factors so it is pivotal for immune system in this complex dynamic process.

Meanwhile, IO also brings concern about IO-related toxicities such as checkpoint inhibitor pneumonitis, colitis, hepatitis, immune checkpoint inhibitors-related endocrinopathies, and dermatologic toxicity.^433–443^ Due to the mechanisms of action, IO especially ICIs, leads to unique toxicity which is different from the toxicities of RT. ICIs can overcome the inhibition of immune cells induced by tumor cells, but it also destroys the status of immune homeostasis which may lead to autoimmunity and nonspecific inflammation. This damage can occur in almost all organs and bring adverse events. ICIs-associated pneumonitis has been widely reported and it may be potentially lethal.^444^ The incidence of ICIs-associated pneumonitis ranges from 3% to 5% in different tumor types.^434^ Although the mechanisms remain unclear, there are three potential pathways which may account for ICIs-associated pneumonitis: generalized immune activation, pre-existing autoantibodies, and off-target effects.^428^ Another worrisome adverse event of IO is endocrine toxicities of ICIs involving the thyroid, pituitary, adrenal, and pancreas.^437^ The potential mechanisms of ICIs-associated endocrine toxicities are similar to ICIs-associated pneumonitis. Hypophysitis is rare in the population who have not received ICIs but hypophysitis occurs up to 10% in the patients who receive anti-CTLA-4 therapy.^445^ Pituitary glands also expressed CTLA-4 and these may become the targets of CTLA-4 antibody which explain the high incidence of hypophysitis in the patients receiving CTLA-4 blockades. Moreover, ICIs may lead to hypothyroidism, hypocortisolism and diabetes mellitus due to the toxicities of ICIs on respective organs.

It is an unquestionably important one mechanistically to evaluate the safety of iRT. For instance, radiation recall pneumonitis, an entity described as pneumonitis localized to a previously irradiated field after exposure to a systemic agent, may occur when using iRT and recently there are several case reports focusing on radiation recall pneumonitis induced by ICIs.^446–450^ Teng et al. proposed that ICIs may lead to an inflammatory response in patients’ regions receiving previous RT, with the process of lymphocytes infiltration and cytokines release which may reveal the mechanism of radiation recall pneumonitis.^451^ Thus, these adverse events increase significant concern for overlapping pulmonary toxicity of iRT. However, there is no evidence to suggest that toxicities of iRT are overtly higher than those with ICIs or RT alone. The currently available clinical trials suggest that iRT is likely to be well tolerated with acceptable toxicity in patients with different tumor types and there are many ongoing clinical trials to explore this issue.^42,367,452–459^ Furthermore, a recent study reported that concurrent nivolumab, ipilimumab, and SBRT were not more toxic than sequential therapy and multi-site SBRT was well tolerated in widely metastatic patients.^412^ Summarizing the available evidence to date, we observe that iRT may result in grade 1 to 2 toxicity generally but the occurrence of toxicity necessitating medical support (grade 3) or which is life threatening (grade 4) is relatively rare. The rate of observed toxicities varies markedly for several reasons, including patient selection and clinical characteristics and heterogeneity of therapies. More clinical data are required to balance the survival benefit and normal tissue toxicities of iRT.

## Prospects

Based on the immune effects and immunosuppressive effects induced by IR, this review elaborates the preclinical rationales of iRT and its clinical results. In view of the abscopal effect, these data indicate that iRT might be a novel regimen for patients with locally advanced tumor especially oligometastatic tumors. Furthermore, investigations about multi-site radiation and radscopal effect of low-dose radiation, and the sense that RT triggers a systemic antitumor response with the help of ICIs and low-dose radiation, seem to be promising. Critically, before the widespread application of iRT as a systematic treatment in clinical practice, we expect more investigation to explore this field and overcome the challenges discussed above.
